# Structural and functional development of twelve newly established floodplain pond mesocosms

**DOI:** 10.1002/ece3.8674

**Published:** 2022-03-08

**Authors:** Sebastian Stehle, Alessandro Manfrin, Alexander Feckler, Tobias Graf, Tanja J. Joschko, Jonathan Jupke, Christian Noss, Verena Rösch, Jens Schirmel, Thomas Schmidt, Jochen P. Zubrod, Ralf Schulz

**Affiliations:** ^1^ Eusserthal Ecosystem Research Station University Koblenz‐Landau Eusserthal Germany; ^2^ iES Landau Institute for Environmental Sciences University Koblenz‐Landau Landau Germany; ^3^ 549911 Federal Waterways Engineering and Research Institute Karlsruhe Germany; ^4^ 549911 Zubrod Environmental Data Science Landau Germany

**Keywords:** ecosystem development, ecosystem function, freshwater colonization, primary succession, principal component analysis

## Abstract

Ecosystems are complex structures with interacting abiotic and biotic processes evolving with ongoing succession. However, limited knowledge exists on the very initial phase of ecosystem development and colonization. Here, we report results of a comprehensive ecosystem development monitoring for twelve floodplain pond mesocosms (FPM; 23.5 m × 7.5 m × 1.5 m each) located in south‐western Germany. In total, 20 abiotic and biotic parameters, including structural and functional variables, were monitored for 21 months after establishment of the FPMs. The results showed evolving ecosystem development and primary succession in all FPMs, with fluctuating abiotic conditions over time. Principal component analyses and redundancy analyses revealed season and succession time (i.e., time since ecosystem establishment) to be significant drivers of changes in environmental conditions. Initial colonization of both aquatic (i.e., water bodies) and terrestrial (i.e., riparian land areas) parts of the pond ecosystems occurred within the first month, with subsequent season‐specific increases in richness and abundance for aquatic and terrestrial taxa over the entire study period. Abiotic environmental conditions and aquatic and terrestrial communities showed increasing interpond variations over time, that is, increasing heterogeneity among the FPMs due to natural environmental divergence. However, both functional variables assessed (i.e., aquatic and terrestrial litter decomposition) showed opposite patterns as litter decomposition rates slightly decreased over time and interpond differences converged with successional ecosystem developments. Overall, our results provide rare insights into the abiotic and biotic conditions and processes during the initial stages of freshwater ecosystem formation, as well as into structural and functional developments of the aquatic and terrestrial environment of newly established pond ecosystems.

## Graphical abstract

We report findings on the initial phase (i.e., 21 months) of ecosystem development for twelve floodplain pond mesocosms located in south‐western Germany. Principal component analyses revealed season and succession time (i.e., time since ecosystem establishment) to be significant drivers of changes in environmental conditions. Initial colonization of both aquatic and terrestrial pond ecosystem components occurred within the first month, with subsequent season‐specific increases in richness and abundances for aquatic and terrestrial taxa, as well as increasing heterogeneity among the ponds, over the entire study period.

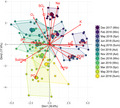

## 1 INTRODUCTION

Ecosystems are characterized by abiotic and biotic components that are linked by multiple processes and through nutrient cycles and energy flows. These systems are dynamic and developing entities controlled by external and internal factors. Mature (climax) ecosystems, developed over long‐term periods, are often complex and relatively stable systems. In contrast, ecosystems at their initial developmental stages are far less complex and stable, with fewer biotic and abiotic components interacting, and characterized by high levels of stochasticity (Buma et al., 2019; Fath et al., 2004). The period between the start of ecosystem development (i.e., the “point zero”) and the quantitative establishment of a first dynamic equilibrium of element cycling can be defined as initial ecosystem stage (Elmer et al., 2013; Schaaf et al., 2011). Such initial systems are more homogenous and less structured than mature ecosystems, which are the results of previous ecosystem succession (Elmer et al., 2013; Odum, 1969). Generally, succession as an important concept in ecology encompasses the directional and continuous occurrence of a range of successional sequences within an ecosystem over varying time scales (Begon et al., 2006). With ongoing succession, the ecosystem complexity increases, with “opportunist” pioneer, short‐lived species colonizing the ecosystem during initial stages (Connell & Slatyer, 1977), which are replaced at later stages by new erratic species with more passive colonization abilities (Ruhí et al., 2013). In addition to changes in community composition, also biomass, interactions, respiration, and information (e.g., genetic diversity) increase during ecosystem development, whereas entropy decreases (Fath et al., 2004; Odum, 1969).

Natural ponds as abundant and ecologically important freshwater ecosystems in Europe and globally (Downing et al., 2006; Kristensen & Globevnik, 2014) have been in the focus of succession research for more than a century (e.g., Allee, 1911). One prominent example is the Pinkhill Meadow pond complex, consisting of approximately 40 permanent, semipermanent and seasonal ponds in the River Thames Floodplain, Oxfordshire, UK. Selected ponds of this complex have been monitored since their construction in 1990 (Freshwater Habitats Trust, 2021). However, the knowledge on the abiotic conditions, biotic communities, and initial ecological processes, including their drivers, of newly established pond sites is generally limited (Miguel‐Chinchilla et al., 2014; Williams et al., 2008). It is rarely possible to study the “point zero” of ecosystem development and primary succession in newly established ecosystems under natural conditions (Schaaf et al., 2011). Most ecological research is conducted in established, climax pond ecosystems, without investigating the abiotic and biotic conditions and processes that led from the initial stages to the development of mature systems (Raab et al., 2012). Also, available information on key environmental factors, such as physico‐chemical water quality parameters and seasonality, are limited leaving a knowledge gap in what is driving primary succession and ecosystem development in newly established ponds (Williams et al., 2008). For this reason, monitoring of pond ecosystem development from the “point zero” provides valuable information on factors driving ecosystem structuring and functioning at initial development stages.

The colonization and subsequent community development during primary successional stages in new pond habitats is determined by stochastic primary effects, local environmental conditions, and biological interactions (Buma et al., 2019; Weidlich et al., 2021; Wiegleb et al., 2017). Along the initial structural developments, also ecosystem functioning evolves driven by the functional traits of the organisms present. The differences in aquatic taxa composition directly affect ecosystem processes and consequently functioning (de Bello et al., 2010; Herbert et al., 2016). Ecosystem functions such as litter breakdown (Gessner & Chauvet, 2002) also denote suitable indicators for the primary successional development and integrity of young emerging ecosystems.

In this study, we conducted an ecosystem primary succession monitoring considering a variety of physico‐chemical parameters, aquatic and terrestrial taxa as well as variables to assess ecosystem functioning. This was done to investigate pond ecosystem developmental trajectories during 21 months from their establishment. The monitoring was conducted in a system of twelve natural floodplain pond mesocosms (FPM 1–12, natural and undisturbed lotic freshwater ecosystems planned and constructed at a research site and operated without anthropogenic manipulation) that have been established in 2017 at the Eußerthal Ecosystem Research Station (EERES; see https://www.uni-koblenz-landau.de/de/landau/fb7/umweltwissenschaften/eeres) in south‐western Germany. The FPMs used here represent systems at “point zero” of ecosystem development and enable repeated sampling and analyses of initial development stages in a fully replicated way. We used ordination methods (i.e., principal component analyses [PCA] and redundancy analyses [RDA]) to analyze pond environmental conditions and interpond variations during initial successional stages.

Besides insights into primary succession and initial pond ecosystem developments, our study contributes to the knowledge of experimental mesocosm studies used to disentangle stressor effects from other confounding factors (e.g., Finnegan et al., 2018; Hua & Relyea, 2014), as aquatic communities of these systems also generally are in very early stages of succession. Insights into primary succession of pond mesocosms, factors driving aquatic community development, and lotic ecosystem development trajectories as gained here may benefit the interpretation of findings from other studies focusing on artificially stressed mesocosm experiments. The objectives of this paper are (1) to monitor and describe the ecosystem development of twelve FPMs using 20 physico‐chemical and biotic parameters; (2) to disentangle and analyze factors driving the development of environmental conditions within the pond ecosystems; and (3) to ascertain interpond variations (i.e., differences among ponds by natural heterogenization) over time.

## 2 METHODS

### 2.1 Study site and FPMs

This study has been conducted at the EERES located in Rhineland‐Palatinate, south‐west Germany (49°15′14″N, 7°57′42″E). The EERES site is situated in a small valley of the Franco‐German Palatinate Forest‐North Vosges UNESCO biosphere reserve, which is an extensively forested low‐mountain range. In 2017, twelve FPMs have been constructed at the EERES site (see Figure S1 for an overview). Each FPM is connected to a small stream called Sulzbach via controllable inlets and overflows. The inlets, except for rare events (see below), were kept closed during the study period preventing the water from the Sulzbach to mix with pond water. The Sulzbach belongs to the stream type “small, fine‐substrate dominated, siliceous highland rivers” (Westermann et al., 2011). The upstream catchment of the Sulzbach has only minor anthropogenic influences and the stream is characterized by a high structural and ecological quality. The FPMs are orientated in east (inflow)–west (outflow) direction. Each FPM has a dimension of 23.5 m × 7.5 m with V‐shaped banks at three sides and a flat water–land floodplain area at the inflow (Figure S2a). The depths of the FPMs increase towards the outflow area (bed slope 1:20), providing a maximum water storage level of 1.5 m. The banks and the bottoms are impervious to water due to a PVC membrane covered by a sand layer of 15 cm; an additional 0.5 m gravel layer (uniform gravel diameter of >2 cm) covers half of each FPM pond bottom toward the outflow area (Figure S2a). The upper half of the banks was seeded with a standard grass mixture (*Festuca rubra* ssp., *Festuca trachyphylla*, *Poa pratensis*) in October 2017 to prevent erosion. After flushing the newly constructed FPMs twice with water from the Sulzbach, the ponds were filled with Sulzbach water on 04 November 2017 to a water level of 30 cm at the outlet sites (Figure S2b). This water level was kept constant at 30 cm (±10%) during the entire study period, meaning that losses due to evaporation were compensated by refilling with water from the Sulzbach. However, refilling of the FPMs was needed rather infrequently (i.e., approximately every 2–3 months depending on meteorological conditions). During the refilling, in order to ensure reproducibility, the inlets of all FPMs were always opened at the same time and for the same brief (i.e., approximately 2 h) duration. Given that the volume of the inflow water was small compared with the water volume of the ponds and that all FPMs received water from the same stream, we estimate the influence of the stream water on the abiotic environmental conditions of the FPMs as marginal. Apart from refilling the ponds, no water inflow into the FPMs occurred. The FPMs thus represent small lentic water bodies with water–land floodplain areas. It has to be noted that the water from the Sulzbach was not sterilized before it was used to initially fill or to refill the ponds. However, during the entire experimental phase, no anthropogenic manipulation or management of the twelve FPMs took place to allow natural primary succession and undisturbed development. The EERES research station is completely fenced off to avoid disturbances by human visitors or larger wild animals. Since October 2019, the FPMs are a key research site of the DFG Research Training Group 2360 “SystemLink” (https://systemlink.uni‐landau.de).

### 2.2 Overview of the monitoring program and sampling methods

A monitoring program was launched immediately after the establishment of the FPMs. The initial ecosystem development and primary succession of the twelve FPMs from a defined “point zero” was monitored for 21 months from 06 November 2017 until 31 July 2019. Overall, 20 variables were surveyed: four parameters describing the general conditions at the EERES site and of the FPMs; five physico‐chemical water quality parameters; nine biotic parameters; two functional parameters. Together, the 20 variables surveyed describe both the aquatic and adjacent terrestrial FPM ecosystem development (Table S1). Because of the high workload, it was not feasible to achieve the same temporal coverage and sampling dates for all parameters during the monitoring; not all parameters were surveyed regularly and during the entire study period. Specifically terrestrial species (i.e., terrestrial vegetation, ground beetles, leafhoppers) were assessed at a few distinct sampling dates only (see details on sampling design below). For this reason, absence of values in the figures and tables correspond to absence of data. During sampling campaigns, all sampling and measurement equipment was completely rinsed with tap water after usage in one FPM to prevent the transfer of organisms or biological material between the ponds. In addition, all sampling and measurement equipment (e.g., polyethylene mesh bags, data logger) were leached for at least 24 h in tap water (with at least three water exchanges) before it was placed in the FPMs in order to prevent leaching of chemicals into the water.

The information on meteorological conditions, soil temperature, and water level fluctuations can be found in the Appendix S1 (supporting results). To track visible real‐time changes during the FPM development, we recorded a time‐lapse video of FPM9 using a daily camera trap (Bushnell Trophy Cam HD) (available at https://youtu.be/vfTNpwyYhFE).

### 2.3 Physico‐chemical water quality parameters

Water samples were taken monthly or bimonthly in each FPM 5–10 cm below the water surface using high‐density polyethylene bottles. Water samples were analyzed by an accredited laboratory applying standard analytical methods (AGROLAB, Germany) for the following components: total organic carbon (TOC), dissolved organic carbon (DOC), fluoride, chloride, nitrite, nitrate, phosphate, sulfate, ammonium, sodium, potassium, calcium, and magnesium. The limits of quantification (LOQ) are given in Table S5. Please note that due to analytical problems (i.e., equipment failure), not all components could be analyzed at all sampling dates. Detected concentrations (i.e., concentration > detection limit) less than the LOQ were replaced by 0.5 × LOQ for data evaluation and visualization.

Specific conductivity and pH were measured monthly or weekly 5–10 cm below the water surface near the outlet sites of the FPMs using a portable analytical device (WTW Multi 3630 IDS). No data for conductivity are available for January 2019.

Water temperature and dissolved oxygen were recorded by a miniDOT^®^ logger (miniDOT^®^ USB Oxygen Logger 7392) installed 5 cm above the bottom gravel layer (i.e., at 25 cm water depth) near the outlet site of each FPM. The miniDOT^®^ logger recorded the water temperature and dissolved oxygen with a resolution of 10 min; daily averages of water temperature and dissolved oxygen were used for evaluations. Monitoring results for all physico‐chemical water quality parameters are presented in the Appendix S1 (supporting results).

### 2.4 Aquatic macroinvertebrates

Benthic macroinvertebrates were sampled 15 times over the sampling campaign using pebble baskets as artificial substrates (see e.g., Brock et al. (1992) for details). Pebble baskets consist of a polyethylene mesh (length 15 cm; width 15 cm; height 7.5 cm) formed as a pyramid and were filled with the bottom substrate (gravel with a diameter of >2 cm) of the FPM. While the upper part of the pebble basket was made of a coarse mesh (2 cm aperture) to enable easy colonization, the lower section was made of a fine mesh of 0.5 cm aperture to prevent escaping of macroinvertebrates during sampling. In each pond, a pebble basket was placed at the bottom near the inlet and outlet sites, as well as in the middle of each FPM, given a total of three baskets per FPM. Before animal collection, baskets were left for colonization for three weeks. At the end of each sampling campaign, the pebble baskets were gently retrieved from each FPM with a dip net (500 µm mesh aperture). The macroinvertebrates present on the pebble basket substrates were removed and preserved in ethanol (70% vol.). All individuals were subsequently identified to the family level.

### 2.5 Merolimnic insect emergence

We sampled the emergence of merolimnic insects from April to July 2019 using three floating pyramidal tents with a basal area of 0.25 m^2^ as emergence traps (Cadmus et al., 2016). The emergence traps were made of nylon mesh and anchored to the pond bottom. Emergence traps were installed from 24 April 2019 until 31 July 2019 and emptied once per week, that is 15 times in total. Three emergence traps per pond were placed at the water surface at locations comparable to the pebble basket sites. Emerged insects were collected in plastic bottles connected to the top of the trap and filled with ethylene glycol to preserve insects. Upon collection, aquatic insects were stored in ethanol (70% vol.) and subsequently identified to the family level. All results of the merolimnic insect emergence monitoring are available in the Appendix S1 (supporting results).

### 2.6 Zooplankton

Zooplankton was sampled monthly in each FPM with a plankton net (20 cm diameter; 65 µm mesh aperture). Overall, 15 samples were taken in all FPMs during the study period, with no samples available for January and February 2018. For sampling, the plankton net was moved gently across 10 m horizontally through each FPM at a distance of approximately 1 m from the banks. After retrieval, samples were rinsed through a net (mesh aperture 120 µm) and stored in ethanol (70% vol.). Zooplankton abundances (individuals >250 µm) were assessed by the Institute for Fishery of the Bavarian State Research Center for Agriculture using the ZooScan V4 system (HydroptiC, version 2.4.0), a digital zooplankton image analysis (Gorsky et al., 2010). Please note that zooplankton individuals <250 µm could not be assessed by the ZooScan digital image analysis.

### 2.7 Amphibians (tadpoles) and submerged vegetation

Amphibians and submerged vegetation, that is, filamentous algae and *Elodea* spec., were assessed by visual inspection of the percentage tadpole coverage and the percentage submerged vegetation coverage, respectively, of the water surface area. Visual inspection for tadpole presence was conducted in spring and early summer (i.e., April—June) 2018 (four times) and 2019 (16 times, observations from end of March until June), that is, during times with tadpole presence. For submerged vegetation, visual inspection was made 30 times at bimonthly or monthly intervals during the study period beginning on 31 January 2018. No distinction could be made regarding living and dead submerged vegetation. During each visual inspection, the presence of tadpoles and submerged vegetation was recorded manually using a layout template; the layout templates were subsequently digitized and the percentage areas covered by tadpoles and submerged vegetation, respectively, were subsequently analyzed by image recognition software (Adobe Photoshop version 21.2). In brief, we determined the total number of pixels for the entire surface area of each FPM and the pixel number of the areas covered by tadpoles or submerged vegetation. The percentage areas covered were then calculated by dividing the number of pixel of areas covered by tadpoles or submerged vegetation by the total number of pixels of the entire surface area of the respective FPM.

### 2.8 Crayfish and fish

The presence of crayfish, which may dominate ecosystem development (e.g., Reynolds et al., 2013), in the FPMs were monitored using three baited standard crayfish traps (type: “Pirate“) per FPM. Overnight monitoring was conducted monthly between April and October in 2018 and between March and July in 2019. The presence of fishes was also checked in the crayfish traps, as well as by visual inspection during each visit of the FPMs. No crayfish or fish were found during the entire study period.

### 2.9 Terrestrial vegetation

The terrestrial vegetation was recorded twice by visually inspecting the northern and southern banks as well as the terrestrial part of the floodplain area of each FPM. The vegetation surveys took place in November 2018 and May 2019.

### 2.10 Ground beetles

Ground beetles were collected by pitfall traps (Schirmel, 2020) during seven sampling campaigns conducted in late spring and autumn 2018, as well as in late spring 2019. One pitfall trap was placed at the northern and southern bank of each FPM and two pitfall traps at a distance of two meters from the shore into the terrestrial part of each floodplain area. The pitfall traps consisted of a plastic cup (6.5 cm diameter) filled with ethylene glycol diluted 1:3 with water and a drop of detergent. At the end of each sampling campaign, the pitfall traps’ contents were sieved and the ground beetles were stored in ethanol (70% vol.). Ground beetle individuals were subsequently identified to species level.

### 2.11 Leafhoppers

Leafhoppers were surveyed on 26 June and 21 September 2019 at the northern and southern banks of FPM3, FPM5, FPM8, FPM10. Leafhopper collection was conducted by suction sampling with a modified leaf blower (SH 56 Stihl, Waiblingen, Germany) using 80 suction pulses per sampling (Kormann et al., 2015). The leafhopper catches were transferred into a bucket and subsequently separated from the rest of the catch with an aspirator. Leafhopper samples were then stored in ethanol (70% vol.) and identified to species level.

### 2.12 Aquatic and terrestrial litter decomposition

Aquatic leaf litter decomposition was approximated using the litterbag method (Benfield et al., 2017). In brief, coarse‐ (10 mm aperture) and fine‐mesh (1 mm aperture) litterbags were filled with approximately 4 and 2 g (weighed to the nearest 0.001 g), respectively, of oven‐dried (at 60°C for 24 h) alder leaves (*Alnus glutinosa* (L.) Gaertn.) (Voß et al., 2015). Fine‐mesh and coarse‐mesh bags were employed in the FPMs to separate the microbially and the shredder‐mediated share of leaf litter decomposition. One coarse‐mesh and one fine‐mesh litterbag were submerged near the inlet and outlet sites, as well as in the middle part of each FPM for 20–25 days at nine dates between May 2018 and June 2019. After retrieval, the remaining leaf material was gently rinsed under running water to remove mineral particles and macroinvertebrates, oven‐dried at 60°C for 24 h and reweighed to the nearest 0.001 g.

The litter decomposition rate *k* was calculated for each FPM and the respective sampling dates using the following formula (Benfield et al., 2017):

$$k=\frac{‐\ln\left(\frac{S(t)}{S(0)}\right)}{t}$$

where *S*(*t*) is the leaf mass as a function of deployment time *t* and *S*(0) is the initial mass of the coarse‐ and fine‐mesh litterbags, respectively. The mass data from the fine litterbags were used to calculate microbial decomposition (*k*_microbial_). For the calculation of the litter decomposition by shredders (*k*_shredders_), *S*(*t*) of the coarse litterbag at a given sampling site was corrected for the mass losses due to microbial decomposition by adding to *S*(*t*) the amount degraded over time in the coarse litterbags (i.e., *S*(0) − *S*(*t*)) multiplied by the mean percentage microbial decomposition of the respective fine litterbags. For data evaluation, decomposition rates were averaged across the three sampling locations of a given pond.

The terrestrial litter decomposition was assessed using the tea bag approach (Keuskamp et al., 2013). In brief, one green tea and one rooibos tea bag (brand “Lipton”) were buried pairwise at a depth of five cm at the middle of each the north and south banks (50 cm above water surface) of each FPM for approximately 21 d at twelve dates during the study period. After retrieval, adhered soil particles were removed and the filling of the tea bags was oven‐dried for 24 h at 60°C and weighed to the nearest 0.001 g. The terrestrial litter decomposition was calculated as linear weight loss per day (mg/d) for both green and rooibos tea as follows:

$$\text{Linear}\;\text{weight}\;\text{losss}\;\text{per}\;\text{day}=\frac{\left(M_{R}‐M_{t}\right)}{t}$$

where *M_R_* is the reference mass derived from averaging the weight of ten green and rooibos tea bag fillings, respectively, and *M_t_* is the mass of the green and rooibos tea bag fillings after deployment time *t*. The mean value of the north and south bank sampling sites of a given FPM was calculated separately for green and rooibos tea bags, respectively, and used for data evaluation.

### 2.13 Generalized additive models for temporal trend analyses

To visualize and assess overall temporal trends in biotic communities and ecosystem function, we fitted generalized additive models (GAM) using the mgcv R package (Wood, 2011). We fit models for the following variables across all FPMs as functions of time since establishment: the number of macroinvertebrate families, the number of zooplankton individuals per sample, the percentage coverage by submerged vegetation, the aquatic litter decomposition rates *k*_microbial_ and *k*_shredders_, and the terrestrial litter decomposition expressed as linear weight loss per day for green tea and rooibos tea. In most cases, we used a Gaussian residual distribution and ten basis functions. For the two models on the number of macroinvertebrate families and the number of zooplankton individuals, we used a negative binomial residual distribution and for the latter 19 basis functions. For the model on *k*_microbial_ and *k*_shredders_, we used nine and three basis functions, respectively. Residual diagnostics were performed visually and the number of basis functions was evaluated with the test proposed in Wood (2017), available through the *gam.check()* function.

### 2.14 PCA and RDA of pond ecosystem developments

We used PCA and RDA ordination analyses to analyze aquatic ecosystem development and environmental conditions of the twelve FPMs and to assess interpond environmental diversities. Briefly, PCA allows to reduce the dimensionality of the data set of environmental variables by explaining the correlation among a large number of environmental variables in terms of new orthogonal, uncorrelated variables (principal components [PCs]) without losing much information (Olsen et al., 2012; Ramette, 2007). Using multiple linear regression, RDA extends PCA by explaining variation between independent and dependent variables within an iterative process to find the best ordination (Ramette, 2007). The following environmental variables (month scale) were included in the PCA and the RDA: specific conductivity, pH, dissolved oxygen, water temperature, DOC, fluoride, chloride, nitrate, phosphate, sulfate, sodium, ammonium, potassium, magnesium, calcium, water level, and submerged vegetation as an indicator of the habitat structure of the FPMs (Caquet et al., 2001; Christman et al., 1994). All other environmental variables were excluded as they were either not directly related to the analysis of the aquatic ecosystem development and pond environmental conditions (e.g., terrestrial vegetation) or had an excess in number of missing values (e.g., tadpole coverage, leaf litter decomposition) across the entire study period (see Table S13 for a complete list of excluded variables). We conducted full PCAs and RDAs, respectively, for the entire study period, as well as separate PCAs and RDAs for each year, that is, for December 2017–June 2018 (first year), and for December 2018–June 2019 (second year); data for noncomparable months were excluded from PCAs for individual years. PCAs and RDAs were conducted both for months/seasons for temporal primary succession analyses, as well as using grouping of ponds to ascertain pond environmental diversities. We used stepwise forward selection in RDA and conducted additional variance partitioning analyses (VPA; e.g., Dray et al., 2012). Data treatment included removing of n/a, checking and removing of outliers, as well as logarithmic (log_10_ + 1) data transformation. Assumptions (e.g., linearity, homogeneity of variances, multicollinearity, residuals) for PCA and RDA were checked prior to analyses and selection of PCA axes was done using the broken stick approach (MacArthur, 1957). All statistical analyses were done using R (version 4.0.2).

## 3 RESULTS

### 3.1 Colonization of aquatic and terrestrial ecosystem compartments

#### 3.1.1 Aquatic macroinvertebrates

**FIGURE 1:**
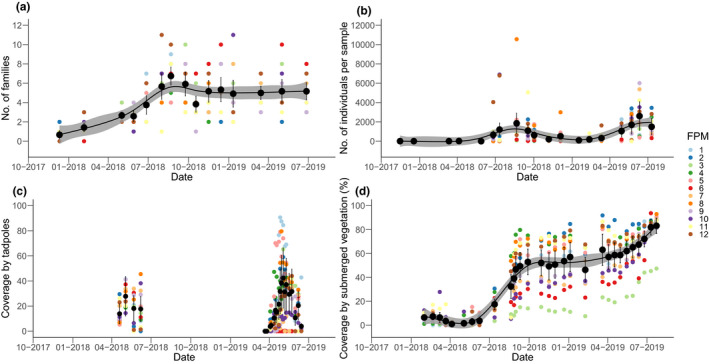
Generalized additive model (GAM) plots for (a) number of macroinvertebrate families and (b) zooplankton population (no. of individuals per sample) in the FPMs over time. (c) Percentage coverage of FPM 1–12 by tadpoles in spring/summer 2018 and 2019, and (d) GAM plot for percentage coverage of the FPMs by submerged vegetation over time. Black circles depict means and error bars show 95% confidence intervals. The gray shaded areas in (a), (b) and (d) indicate the 95% confidence intervals

Overall, 7998 individuals belonging to 35 macroinvertebrate families and seven orders were identified in the twelve FPMs during the study period. The temporal trajectory for family richness shows increasing richness from December 2017 (mean of 0.7 families; 95% CI [0.24, 1.09]) until the end of August 2018 with a mean of 6.8 (95% CI [5.82, 7.68]) families across the FPMs (Figure 1a). After a decrease in family richness until October 2018, family richness leveled off until the end of the study period at around a mean of five families per FPM. GAM indicated a significant temporal trend for family richness development across all FPMs (*p* < .001; Figure 1a). However, there were large variations in family richness among FPMs at the individual sampling dates, particularly from summer 2018 onwards (Figure 1a). A maximum of 11 macroinvertebrate families per individual sampling was recorded for FPM12 (August 2018) and FPM10 (January 2019).

Variation was found also among ponds, across the study period; most of the macroinvertebrate families (*n* = 19) were found in FPM12 and 17 families in FPM1, FPM3, FPM6, FPM7, FPM9; in contrast, only eleven families were identified in FPM11 (Table S7). Concerning abundances, most individuals were found in FPM3 (*n* = 1052) and FPM12 (*n* = 1494), whereas FPM5 (*n* = 332) had the lowest abundances (Table S7).

#### 3.1.2 Zooplankton

The trajectory of zooplankton population size shows fluctuations over the study period. Zooplankton population size in the FPMs was low (i.e., <100 individuals per sample) until the end of June 2018 (Figure 1b; Table S7). Abundances subsequently increased reaching a peak at the end of August 2018 with an average of 1852 (95% CI [320, 3384]) individuals per sample across all FPMs. After a subsequent decrease to a minimum in January 2019 (mean of 100 (95% CI [43, 157]) individuals per sample across FPMs), zooplankton abundances increased again to a maximum in mid‐2019 (mean of 2607 (95% CI [1585, 3628]) individuals per sample across FPMs). GAM analysis indicated the overall temporal trend of zooplankton population sizes in the FPMs to be significant (*p* < .001; Figure 1b).

Considering individual ponds, large differences in zooplankton abundances were found between FPMs at individual sampling dates specifically during late spring and summer months, while differences were clearly smaller in autumn and winter months (Figure 1b). In August 2018, FPM8 showed an exceptionally high abundance of 10,560 individuals per sample. Zooplankton individual numbers were overall highest in FPM2 and FPM8 to FPM12, whereas FPM3 and FPM6 generally had lowest abundances (Table S7).

#### 3.1.3 Amphibians (tadpoles)

Irrespective of the year of observation, tadpoles were present only from April until June. In 2018, tadpole coverage slightly increased from mid‐April to a maximum at the beginning of May (28% (95% CI [22.8, 32.8]) mean coverage across all FPMs) and then slightly decreased until June 2018 (18% (95% CI [9.5, 25.9]) mean coverage) as adult amphibians migrated from the FPMs (Figure 1c). In 2019, the tadpole coverage followed the same pattern, with, however, a larger increase of tadpole populations until the beginning of May (42% (95% CI [27.7, 56.9]) mean coverage).

Differences in percentage tadpole coverages between individual ponds was higher in 2019 compared with 2018 (Figure 1c); FPM1, FPM5, FPM8 reached maximum coverages of around 80–90% in spring 2019, whereas FPM6, FPM7, and FPM10 had low coverage percentages of <20% (Table S7). Overall, tadpole percentage coverages were on average across all sampling dates highest in FPM1 and FPM5 and lowest in FPM6, FPM7, and FPM10 (Table S7).

#### 3.1.4 Submerged vegetation

The percentage areal coverage of submerged vegetation (i.e., algae and *Elodea* spec.) in the FPMs was low until the end of June 2018 (i.e., generally less than 20% coverage) and then substantially increased in July and August 2018 to around 50% coverage on average across all ponds (Figure 1d). After a phase of stabilization at around 50% coverage during autumn and winter 2018/2019, submerged vegetation coverage increased during spring and summer to generally >80% coverage in July 2019. A significant (*p* < .001) temporal trend for submerged vegetation coverage across all FPMs was indicated by GAM (Figure 1d).

Submerged vegetation coverage was comparable between the individual ponds until August 2018 (Figure 1d; Table S7). However, submerged vegetation development started to differ between the ponds from September 2018 onward, with FPM3 (the only FPM with coverage never exceeding 50%), FPM6, FPM7, and FPM10 continuously characterized by lower submerged vegetation coverages compared to the other ponds. In contrast, FPM2, FPM4, FPM5, FPM8, FPM11, FPM12 showed all above‐average percentage submerged vegetation coverage from September 2018 onward (Figure 1d; Table S7). Apart from FPM3, algae and *Elodea* spec. vegetation coverage of the ponds aligned toward the end of the study period (Figure 1d).

#### 3.1.5 Terrestrial vegetation

**FIGURE 2:**
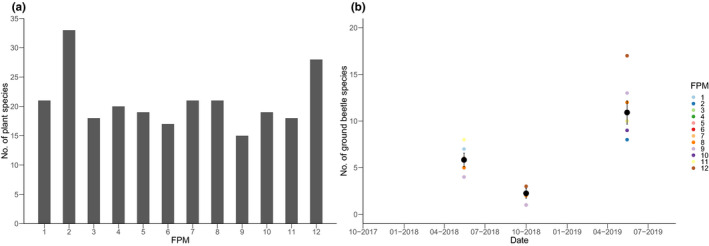
(a) Number of terrestrial plant species identified in November 2018 at the banks and floodplain areas of FPM 1–12, and (b) total number of ground beetle species sampled at the banks and floodplain areas of FPM 1–12 in May 2018, September/October 2018 and May 2019. Black circles in (b) depict means and error bars show 95% confidence intervals

During the first survey in November 2018, 53 grass and plant species were found at the 12 FPMs (see Table S8 for a full list of species). Around 20 plant species were present at each individual FPM, with most species found at FPM2 (33 species) and FPM12 (28 species) (Figure 2a). Eight out of the 53 plant species were present at all FPMs (Table S8). During the second survey in May 2019, only four additional species (i.e., *Luzula* spec., *Vicia sativa*, *Lotus corniculatus*, *Rumex crispus*) were detected.

#### 3.1.6 Ground beetles

Overall, 42 different ground beetle species and 1324 individuals were captured in the adjacent riparian floodplain areas and surrounding banks of the FPMs. The lowest number of species was detected in September/October 2018 (mean number of species across ponds: 2.3; 95% CI [1.7, 2.8]), while the highest number of species was captured in May 2019 (mean number of species across ponds: 10.9; 95% CI [9.7, 12.2]) (Figure 2b).

FPM12 had the overall highest species richness (*n* = 18), as well as the highest richness of one individual sampling date (*n* = 17) (Figure 2b; Table S9); in contrast, only 11 species were detected overall at FPM6. The number of individuals caught during the three sampling campaigns ranged from 71 (FPM2) to 133 (FPM1) (Table S9).

#### 3.1.7 Leafhoppers

Overall, 19 leafhopper species and 910 individuals were sampled in 2018 around FPM3, FPM5, FPM8, and FPM10 (please see Table S10 for a full list of species). The species numbers did not differ much between the four FPMs; however, the number of individuals was twice as high at FPM3 (*n* = 326) compared with FPM8 (*n* = 163) (Table S10).

### 3.2 Aquatic and terrestrial litter decomposition

**FIGURE 3:**
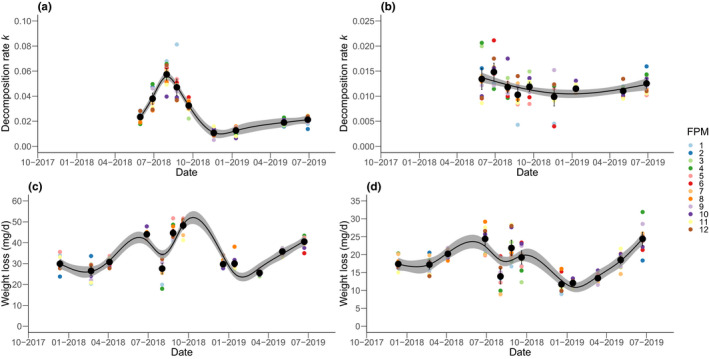
Generalized additive model (GAM) plots for leaf litter decomposition expressed as decomposition rate *k* for (a) microbial decomposition (*k*_microbial_) and (b) shredder‐mediated decomposition (*k*_shredder_) over time. GAM plots for the terrestrial litter decomposition at the banks of the twelve FPMs expressed as linear weight loss per day (mg/day) for (c) green tea and (d) rooibos tea over time. All values in (a–d) are displayed at the end of each sampling period. Black circles depict means and error bars show 95% confidence intervals. The gray shaded areas in (a–d) indicate the 95% confidence intervals

The microbially mediated leaf litter decomposition showed seasonal patterns in all FPMs (Figure 3a). *k*_microbial_ increased from May 2018 (*k*_microbial_ mean across all FPMs: 0.023; 95% CI [0.021, 0.026]) to an overall maximum in July 2018 (*k*_microbial_ mean: 0.057; 95% CI [0.053, 0.062]), followed by a decrease to minimum decomposition values in November 2018 (*k*_microbial_ mean: 0.011; 95% CI [0.009, 0.012]) and January 2019 (*k*_microbial_ mean: 0.013; 95% CI [0.011, 0.014]). Subsequently, *k*_microbial_ slightly increased again until June 2019 (*k*_microbial_ mean: 0.021; 95% CI [0.019, 0.023]) (Figure 3a). The shredder‐mediated leaf litter decomposition differed from the microbial decomposition as it (i) had consistently lower values particularly in summer 2018 and (ii) was rather constant over time, that is, the mean *k*_shredder_ was generally between 0.01 and 0.015 (Figure 3b). GAM results indicate significant overall temporal trends across all FPMs for both decomposition rates (*p* < .001; Figure 3a,b). We found rather small differences (i.e., a factor of 1.1) between *k*_microbial_ and *k*_shredder_ during November 2018 and January 2019 (Figure 3a,b). Both decomposition rates were higher in summer 2018 than in summer 2019.

Considering the entire study period, average decomposition rates of the individual FPMs were comparable, with highest average values for *k*_microbial_ (0.035; FPM1) and *k*_shredder_ (0.014; FPM3) not differing largely from lowest average values for both *k*_microbial_ (0.026; FPM10) and *k*_shredder_ (0.01; FPM11) (Table S11). Nevertheless, larger differences in decomposition rates of the different FPMs existed particularly during the spring and summer months; differences between the ponds were moreover larger in 2018 and less pronounced in 2019 for both *k*_microbial_ and *k*_shredder_ (Figure 3a,b).

Concerning terrestrial litter decomposition, weight losses per day were higher for green tea compared with rooibos tea. However, the terrestrial litter decomposition for all ponds showed a comparable pattern over time for the two different tea bag types, with significant (*p* < .001) overall temporal trends for both parameters identified by GAM (Figure 3c,d). Litter decomposition increased during spring and peaked in summer and early autumn 2018 (highest mean decomposition rates across all FPMs: 48.27 mg/day (95% CI [46.43, 50.12]) for green tea (September 2018) and 24.41 mg/day (95% CI [22.66, 26.16]) for rooibos tea (June 2018)) and decreased in late autumn and during winter months (lowest mean decomposition rates across all FPMs: 25.57 mg/day (95% CI [24.90, 26.24]) for green tea (March 2019) and 11.74 mg/day (95% CI [10.58, 12.89]) for rooibos tea (December 2018)). The weight loss in tea bags was high already at the first sampling date in December 2017 and did not increase when comparing subsequent seasons (Figure 3c,d).

Terrestrial litter decomposition showed generally largest interpond variations during summer and early autumn months, but were overall comparable across the study period (Figure 3c,d; Table S12). Particularly for green tea, the interpond variation was higher in 2018 compared with 2019.

### 3.3 Statistical evaluation of pond ecosystem developments over time

Two PCA axes were selected by the broken stick approach, explaining a total variance of 48.2%. The first PCA axis, which explains 30.6% of the total variance, has significant loadings (correlation > .60) on specific conductivity, potassium, DOC, fluoride, as well as submerged vegetation and pH. The second PCA axis explains 17.6% of the total variance and correlates to sodium and sulfate, as well as to phosphate (Table S13).

**FIGURE 4:**
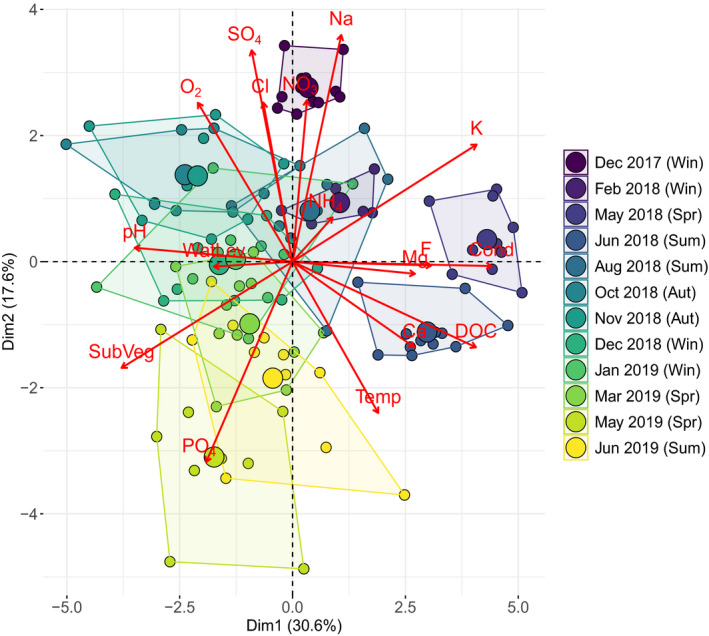
PCA biplot of ecosystem development and environmental conditions in the twelve FPMs for the entire study period. Small color‐coded (see figure legend) dots and polygons represent FPM observations within months (with respective seasons) across the study; larger dots represent the mean for a given month

For the entire study period, no linear pattern of pond environmental condition was identifiable along either the first or second PCs (Figure 4). However, the PCA biplot indicates differences in ordination patterns between the first year (i.e., winter 2017/2018 until summer 2018) and the second year (i.e., winter 2018/2019 until summer 2019) of pond ecosystem development (Figure 4); overall, there were higher differences in pond environmental conditions in the first year compared to the second year. The PCA indicates that the differences in environmental conditions between the different seasons (i.e., winter, spring, summer) are larger in the first year compared with the second year while the interpond diversity in the respective months/seasons was higher in the second year and largest at the end of the study period (i.e., spring and summer 2019). The PCAs performed separately for the first and second year confirmed these results. In the first year, large differences in environmental conditions of the ponds existed among the different seasons (i.e., large seasonal effects), driven by, for example, water temperature and dissolved oxygen (Figure S9a); the interpond variability within each season was, however, small, particularly in winter 2017/2018 and spring 2018. In contrast, the PCA biplot for the second year shows less effects of the seasons in driving overall differences in environmental conditions, while environmental differences among ponds appear to become more influential (Figure S9b). In the same PCA ordination space, when the pond is selected as grouping factor, overall no large differences in environmental conditions are visible among ponds if the entire study period is considered (Figure S10a). Also in this case, when the PCA is constrained to the first and second years, a high similarity of pond environmental conditions is shown until summer 2018 (Figure S10b), whereas differences in environmental conditions among ponds increase substantially in the second year (Figure S10c). This also confirms a lower interpond environmental diversity within seasons (i.e., less seasonal effects) in the second compared to the first year. A further PCA shows that the pond location (i.e., left, middle, right) along the experimental site (see Figure S1) had no effect on environmental conditions of the ponds (data not shown).

The results of the RDA and VPA overall confirm patterns observed in the PCA ordination. RDA and VPA show that for the entire study period successional time (i.e., time since the beginning of the study, VPA explained variance = 36.1%) and its combined effect with seasonality (VPA explained variance = 14.8%) have the largest effect on the environmental conditions of the FPMs (Table 1; Table S14). Similarly to what was observed in the PCA, when the RDA is performed exclusively on the first year of ecosystem development, it identifies season (VPA explained variance = 22.6%) and its interaction with successional time (VPA explained variance = 38.7%) to explain most variance in the environmental conditions of the FPMs (Table 1), whereas the factor pond had no significant influence (Table S15). On the other hand, when the RDA is performed on the second year, analysis identifies pond as significant factor explaining the highest proportion of variance (VPA explained variance = 33.5%) in environmental conditions, followed by the interaction of seasons with successional time (20.8%; Table 1; Table S16).

**TABLE 1:** VPA results for the factors successional time, season, and pond for the entire study period, as well as for the first and second year of the study

Factor	Explained variance (entire study period)	Explained variance (1^st^ year)	Explained variance (2^nd^ year)
Pond	0.062	0.072	0.335
Season	0.045	0.226	0.150
Successional time	0.361	0.087	0.013
Interaction successional time—seasons	0.148	0.387	0.208

## 4 DISCUSSION

### 4.1 Pond ecosystem developments and environmental conditions

Monitoring of the first 21 months of initial pond development and primary succession revealed dynamic ecosystem successional trajectories in the twelve FPMs (see also Figure S2 and the time‐lapse video available at: https://youtu.be/vfTNpwyYhFE). Data from the present survey show that newly established ponds are colonized quickly within weeks or few months; the development of pond ecosystem structure and function is thereby shaped progressively by seasonal influence and the varying abiotic and biotic conditions. The overall initial ecosystem development from the “point zero” observed here for the FPMs is in line with pond studies under field conditions. Similar developmental patterns were, in fact, also observed at a larger scale for the Chicken creek catchment in eastern Germany (Hüttl et al., 2013). Also, our results are comparable to the findings from the Pinkhill Meadow pond complex in the UK, in which a rapid pond colonization and ecosystem development was observed within the first years after construction (National River Authority, 1992).

Fluctuations of physico‐chemical water quality parameters over time (Appendix S1, supporting results) likely resulted from effects caused by seasonality (effects on e.g., water temperature), meteorology (effects of precipitation on e.g., conductivity), and biology (e.g., effects of photosynthesis by aquatic vegetation on pH and dissolved oxygen concentrations). Overall low inorganic ion concentrations are related to the fact that ponds have been entirely filled with water from the Sulzbach, a nutrient‐ and ion‐poor stream. Also, interpond variability of physico‐chemical parameters could be explained by differences either in submerged vegetation coverage (pH values; dissolved oxygen) or differences in soil entries caused by bank erosion (e.g., specific conductivities; ion concentrations). Intersystem variability of physico‐chemical parameters has also been observed in previous studies with replicated outdoor pond mesocosms (e.g., Caquet et al., 2001; Christman et al., 1994). However, care must be taken in interpreting the dissolved oxygen concentration data particularly of FPM9‐12 as there are larger data gaps due to O_2_ logger failures (i.e., defective devices).

PCA and RDA indicate environmental conditions during the successional development of the FPMs to be driven by a resulting effect of succession time, seasonality, and their interaction. The factor successional time was an important driver during the entire study period and particularly during the first year of monitoring, which can be expected for the development of newly established ecosystems after a “point zero” (Miguel‐Chinchilla et al., 2014; Williams et al., 2008). The interaction of successional time with seasonality resulted in changing and nonlinear patterns over time. A large effect of seasonality on the pond environmental conditions during the first year, and environmental homogeneity among the twelve ponds (i.e., low interpond variability) suggests that external environmental cues (e.g., meteorological conditions in the different seasons) were the main drivers for this initial phase of ecosystem development, whereas internal ecological processes within the FPMs played a minor role, resulting in low interpond variation. The seasonal influence on pond environmental conditions decreased in the 2^nd^ year, while the interpond diversity concurrently increased due to natural environmental divergence. External factors (e.g., season‐driven meteorological effects) became thus less important over time, whereas internal factors and processes in the individual ponds led to more diverse ecosystem formations and significant differences in environmental condition and habitat structure; these developments led to increasing complexities and heterogenization of the FPMs. Our findings, comparable to those found in the Chicken creek catchment study (Elmer et al., 2013; Hüttl et al., 2013), confirm that while external factors dominate the very initial phase of ecosystem development, internal interacting abiotic and biotic factors gained importance in later stages. Increases in interactions and feedback, accompanied by, among other, an increase in biomass and species diversity (see below), are clear indicators of ecosystem development and succession (Fath et al., 2004; Odum, 1969).

### 4.2 Pond colonization and aquatic and terrestrial community development

Data from our study show that new, undeveloped ponds are colonized quickly, that is, zooplankton and invertebrate species were recorded in six ponds already at the initial sampling dates (15 November 2017 and 11 December 2017); algae and *Elodea* spec. were found in all ponds during the first survey in January 2018. A fast colonization by aquatic invertebrates and aquatic vegetation was also observed within the first year after construction in four ponds of the Pinkhill Meadow pond complex (National River Authority, 1992; Williams et al., 2008) and in newly created ponds in Wales (Gee et al., 1997). The inherent mobility of freshwater taxa fosters their dispersal and colonization of new habitats (Williams et al., 2008); existing (semi‐)natural ponds and small streams at the EERES site within a distance of less than 100 m from the FPMs likely acted as source habitats for plant and animal propagules colonizing the twelve FPMs. In addition, filling the ponds with water from the Sulzbach most likely contributed to their colonization by actively introducing different taxa such as benthic larvae. At the same time, new pond habitats are characterized by specific environmental conditions, that is, they are dominated by inorganic substrates, have a low coverage of aquatic macrophytes, and lack top predators, such as fish (Williams et al., 2008). Colonizing species and taxa with short generation times such as zooplankton, invertebrates, and algae benefit from such initial conditions and are able to establish populations as observed here. Diverse terrestrial plant communities, as well as leafhopper and ground beetle populations, rapidly colonized the terrestrial component of the FPMs within a few months after the ponds’ establishment with overall more than 100 species. Such a rapid colonization of the terrestrial component of the ponds is in line with colonization patterns of terrestrial plants observed at the Pinkhill pond complex site (Williams et al., 2008). Surveys of the terrestrial communities were not conducted as regularly as the surveys of the aquatic components and did not start directly after the establishment of the ponds. For this reason, drawing conclusions about the very initial colonization phase of the terrestrial banks and floodplain areas of the FPMs is limited.

However, the overall increase of aquatic and terrestrial taxon richness and abundances in all FPMs over the seasons (i.e., higher richness and abundances in subsequent seasons compared to previous seasons) indicates progressive ecosystem development and succession over time. Overall, the colonization and developments of the aquatic and terrestrial communities were comparable between the different ponds, but differences existed in terms of species presence and development of population sizes for multiple aquatic and terrestrial groups, individual sampling dates and across the entire study period. For instance, overall numbers of macroinvertebrate abundances or families of emerged merolimnic insects detected during the study period differed by 450% and 400%, respectively, for individual ponds. The observed increase in variation of the aquatic and terrestrial communities of the FPMs over time also evidences ecosystem development and succession as maturing systems become more structured and less homogenous than systems at earlier stages (Elmer et al., 2013; Fath et al., 2004); the increased biological divergence is in line with the heterogeneity observed in PCA, RDA, and VPA for the environmental conditions (see above), with the physical location of the FPMs not explaining taxa richness and abundances of the ponds. Generally, biological interactions in initial ecosystems are limited so that it is likely that, during the initial phase of development, aquatic communities in the FPMs were shaped by (i) bottom‐up effects induced by habitat quality and the physico‐chemical environments, or (ii) stochastic processes determining species pond colonization. Given that the FPMs are located in close proximity, are similar in size, depth, substrate type, and climatic conditions, and have comparable physico‐chemical characteristics particularly during the first year (see discussion above), differences observed among ponds for taxa richness and abundances during initial ecosystem developments are unlikely to be explained by abiotic environmental conditions (although this relation was not statistically analyzed in this study). Thus, stochastic effects of early FPM species colonization and propagating community effects are more likely explanations for differences in aquatic communities and abundances observed between the FPMs. However, interpond taxa variability (i.e., differences in species numbers and abundances among the FPMs) increased over time, alongside those of abiotic parameters. This likely is a result of ecosystem divergence (see discussion of PCA and RDA results above), priority effects after the stochastic initial phase (Weidlich et al., 2021), as well as increasing biotic–abiotic interactions and feedback in the ponds during their advancing ecosystem development. Street and Titmus (1979) showed for macroinvertebrate communities of six newly constructed experimental ponds that interpond taxa variability was driven by increasing variability of pond environmental conditions and habitat structure (e.g., differences in aquatic macrophyte abundances) within two years of observation. Also, the importance of stochastic sequences of species arrival and subsequent priority effects, in which the initial species assemblages partly determines future community developments, has been largely proven for the colonization of new pond habitats (Chase, 2003; Louette & De Meester, 2007); priority effect‐induced long‐lasting differences in species dominance and overall community assemblages may thus lead to future increases in taxa variability among FPMs. Long‐term pond monitoring in the UK showed larger variations of plant richness and macroinvertebrate richness in ponds after seven years compared to the first year of construction (Williams et al., 2008). Overall, as the FPM communities mature, individual ecosystem properties including abiotic and biotic interactions are expected to increase further in influence over time (Frisch et al., 2012), potentially resulting in further increase of interpond taxa variability.

### 4.3 Pond ecosystem development and ecosystem functioning

Decomposition of plant litter denotes an important ecosystem function for the provisioning of energy and nutrients in aquatic and terrestrial environments (Benfield et al., 2017; Krishna & Mohan, 2017). However, concerning leaf litter decomposition in the FPMs, data were not available before May 2018, so that the aquatic decomposition performances during the very initial stages of ecosystem development could not be determined. Available data, however, show that the leaf decomposition in the FPMs was mostly driven by microbial decomposition processes and less by invertebrate shredder‐mediated decomposition, particularly in summer 2018. This can be explained by the fact that microorganisms (i.e., aquatic fungi and bacteria) have very short generation times, which enables faster colonization and microbiological community growth in the initial pond ecosystems. Moreover, the data indicate that *k*_microbial_ is strongly determined by seasonal effects, that is, leaf litter decomposition is higher during periods with higher water temperatures and lower during colder months. This was expected given that higher water temperatures foster the abundance and activity of microorganisms and thus *k*_microbial_ (Martinez et al., 2014). However, in comparison to *k*_microbial_, *k*_shredder_ was substantially less influenced by seasonal effects, which is possibly explained by the year‐round presence of macroinvertebrates in the FPMs.

In contrast to the increase of numbers of aquatic species and abundances in subsequent seasons, a season‐specific comparison shows that the ecosystem function leaf decomposition decreased over time, which is most pronounced for *k*_microbial_; slightly lower water temperatures in spring and summer 2019 compared to 2018 (Appendix S1, supporting results) might be an explanation. Interestingly, in contrast to what we found for all other abiotic and biotic variables (see above), the differences in leaf litter decomposition rates between the ponds also decreased over time. It follows that this ecosystem function converged with progressing ecosystem developments of the FPMs. One reason for the taxonomical divergence but functional convergence observed here for the second year might be that later arriving species are not yet able to fully compete with species already established in the FPMs after a largely stochastic initial colonization and niches (i.e., energy sources such as litter decomposition) partitioned already between the initial colonizers.

Data from the terrestrial parts of the FPMs in December 2017 revealed terrestrial litter to be efficiently decomposed already under initial ecosystem conditions. Moreover, terrestrial litter decomposition did not increase over time when comparing the respective seasons. This is in line with our findings on aquatic leaf litter decomposition and once again in contrast to our taxonomical findings on the structural ecosystem development, which showed a season‐related increase in aquatic and terrestrial species presence and abundances between the FPMs. That said, ecosystem functions such as aquatic and terrestrial litter breakdown are, however, effective already in the very initial stages of freshwater ecosystem development, whereas ecosystem structure (i.e., diversity and abundance of different taxa) gradually increases over time. However, terrestrial litter decomposition was also strongly influenced by seasonal and meteorological effects, with higher soil temperature and soil moisture fostering an increase in litter decomposition due to elevated microbial activity in warmer months (Krishna & Mohan, 2017). Regarding the latter, the prolonged dry period in July 2018 (Appendix S1, supporting results) may explain the substantial reduction in litter degradation at the beginning of August 2018. The faster decomposition of green tea compared with rooibos tea is well known from the literature and explained by differences in litter composition, particularly cellulose and lignin contents (Didion et al., 2006; Keuskamp et al., 2013).

## 5 CONCLUSION AND OUTLOOK

In our study, the initial freshwater ecosystem development and primary succession could successfully be observed at the EERES site. Using an array of twelve FPMs denotes a novel and promising approach to observe and disentangle abiotic and biotic conditions including structural and functional ecosystem developments as well as their complex interactions. The FPMs at the EERES site offer unique research opportunities to study small freshwater environments, ecosystem development, and water–land interaction under field conditions in a replicated and controlled manner. The ecological research possibilities at the EERES site thus differ from other ecosystem research approaches that either analyze mature ecosystems, or do not feature repeated designs or sites with clear boundary conditions and fully controllable research environments. The findings on ecosystem primary succession and initial ecosystem developments from a “point zero” presented here may benefit planning, implementation, and ecological evaluation of freshwater restoration measurements, which are widely implemented in Germany and Europe (see e.g., http://www.europeanponds.org; https://www.ecrr.org). Also, our monitoring data provide valuable information for the ecological assessment of temporary ponds with seasonal formation in river floodplain areas. Moreover, consequences from anthropogenic disturbances of natural pond ecosystems may become more predictable if fundamental mechanisms of initial ecosystem colonization are better understood. Our findings also support that creating small ponds denotes a simple and cost‐effective tool to enhance landscapes by biodiversity‐rich habitats within short periods of time; this is of particular importance considering the ongoing decline of global freshwater biodiversity (e.g., Albert et al., 2021; Tickner et al., 2020). Last, our study shows that particularly interpond taxa variability among replicated outdoor pond mesocosms in early successional stages must be considered in case these aquatic systems are used to analyze responses to anthropogenic stressors, such as chemicals (Caquet et al., 2000, 2001). Although larger mesocosms such as the FPMs used here are more internally stable and sustainable, which reduces intersystem variability compared with smaller systems (Belanger, 1997; Caquet et al., 2000), larger experimental pond systems also need to be managed (e.g., inserting species into ponds; mixing water and sediments between ponds) to increase replicability between the experimental units.

Future research conducted at the EERES FPMs can range from ecological studies without any anthropogenic manipulation up to entirely controlled and manipulated studies with, for example, highly fluctuating water levels and flooding regimes. The EERES research site is open to future research cooperations in all kind of aquatic and terrestrial research projects. The twelve FPMs of the EERES site are currently used for experimental research of the DFG Research Training Group 2360 “SystemLink” (https://systemlink.uni‐landau.de), which investigates biogeochemical and ecological interactions of aquatic and terrestrial ecosystems under anthropogenic stress.

## CONFLICT OF INTEREST

The authors have no conflict of interest.

## AUTHOR CONTRIBUTIONS

**Sebastian Stehle:** Conceptualization (equal); Data curation (equal); Formal analysis (equal); Methodology (equal); Validation (lead); Visualization (equal); Writing – original draft (lead); Writing – review & editing (equal). **Alessandro Manfrin:** Formal analysis (lead); Methodology (equal); Writing – review & editing (equal). **Alexander Feckler:** Visualization (equal); Writing – review & editing (equal). **Tobias Graf:** Data curation (equal); Investigation (equal). **Tanja J. Joschko:** Conceptualization (equal); Data curation (equal); Funding acquisition (equal); Methodology (equal); Project administration (lead). **Jonathan Jupke:** Formal analysis (equal); Methodology (equal); Writing – review & editing (equal). **Christian Noss:** Conceptualization (equal); Investigation (equal); Writing – review & editing (equal). **Verena Rösch:** Investigation (equal); Writing – review & editing (equal). **Jens Schirmel:** Conceptualization (equal); Investigation (equal); Methodology (equal); Writing – review & editing (equal). **Thomas Schmidt:** Conceptualization (equal); Investigation (equal); Methodology (equal); Writing – review & editing (equal). **Jochen P. Zubrod:** Conceptualization (equal); Investigation (equal); Methodology (equal); Writing – review & editing (equal). **Ralf Schulz:** Conceptualization (equal); Funding acquisition (equal); Methodology (equal); Supervision (lead); Writing – review & editing (equal).

## Supporting information

Appendix S1

## Data Availability

All data of this study are provided in the main text and in the appendix.
