# Supplementary material for: Structural and functional development of twelve newly established floodplain pond mesocosms
Source: Ecol Evol. 2022 Mar 8;12(3):e8674. doi: 10.1002/ece3.8674 (PMC8902662; doi:10.1002/ece3.8674)
Supplement: Supplementary file 1 — Appendix S1 [file ECE3-12-e8674-s001.docx]

**Appendix S1**

**Supporting Methods**

**
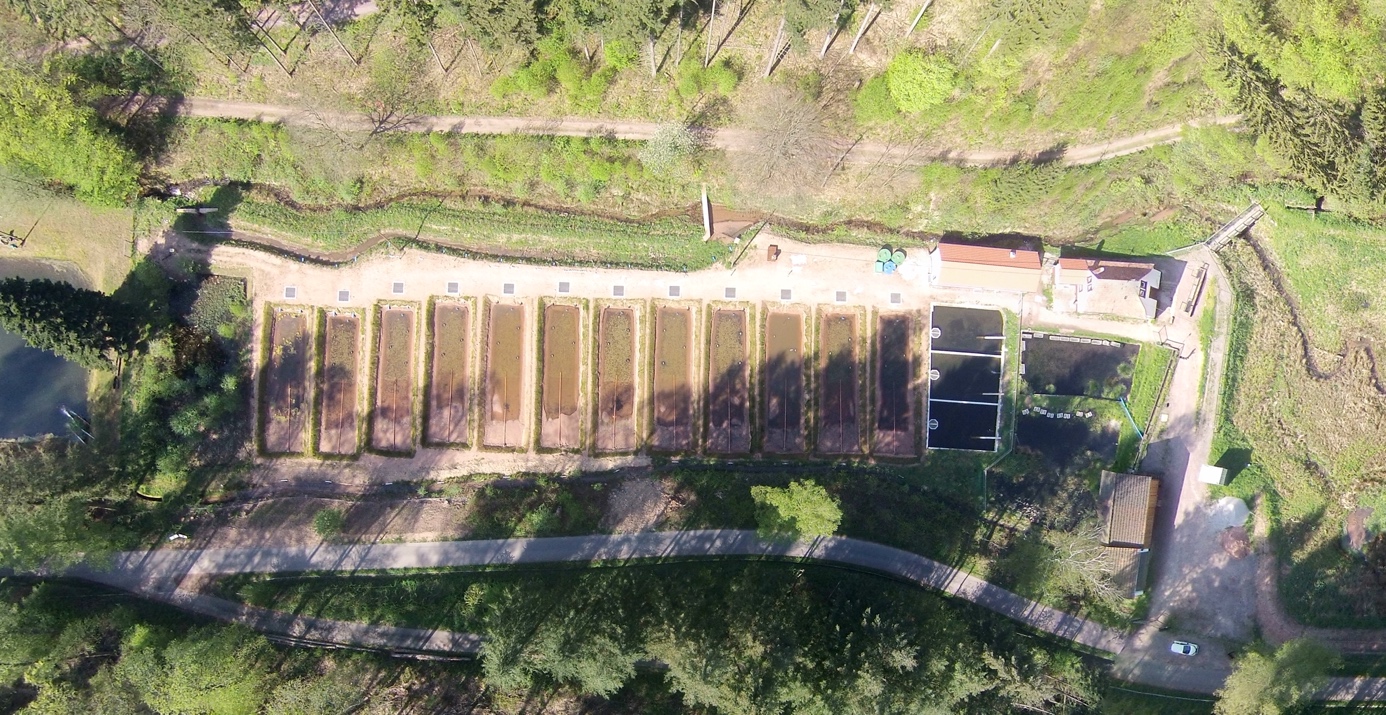
**

**Figure S1.** Aerial image of the twelve floodplain pond mesocosms (FPM 1-12) at the Eußerthal Ecosystem Research Station (EERES). Aerial photo taken in April 2018.


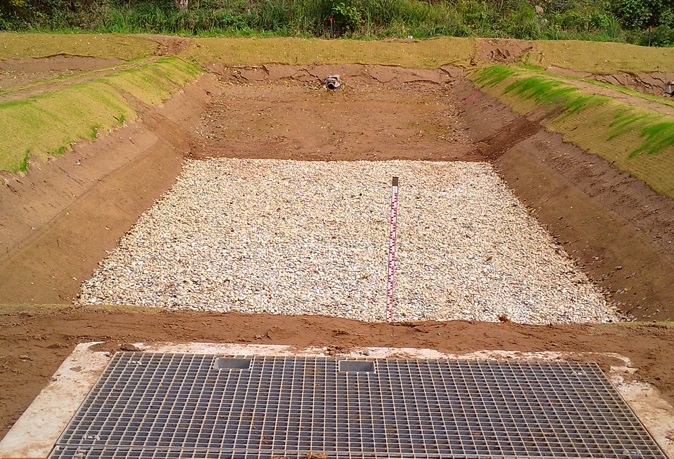

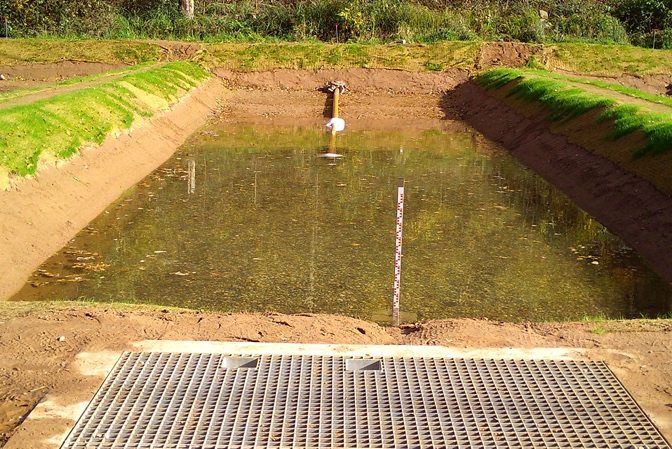


D

C

B

A


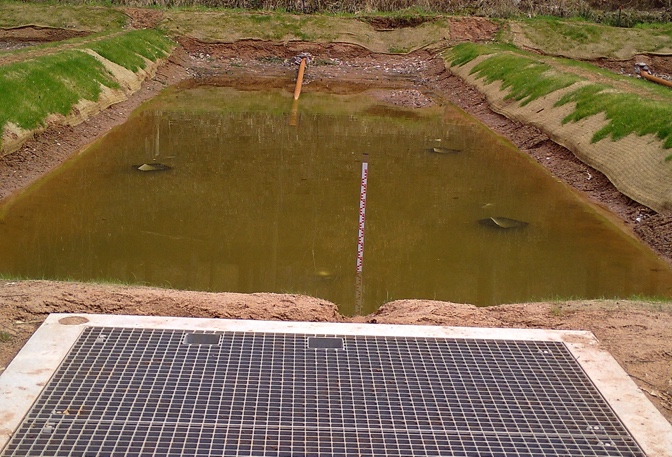

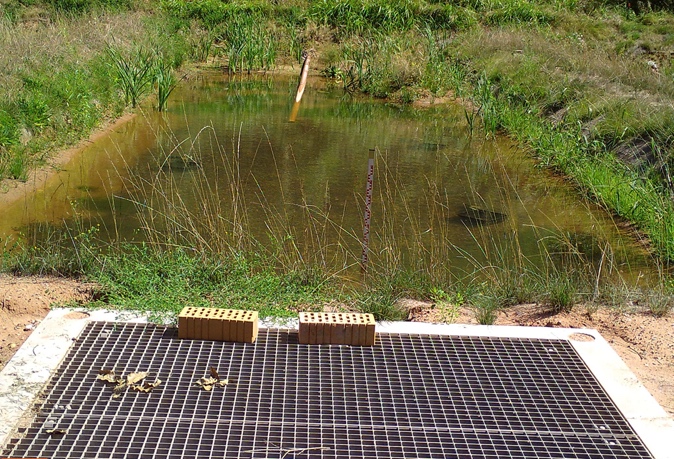


E


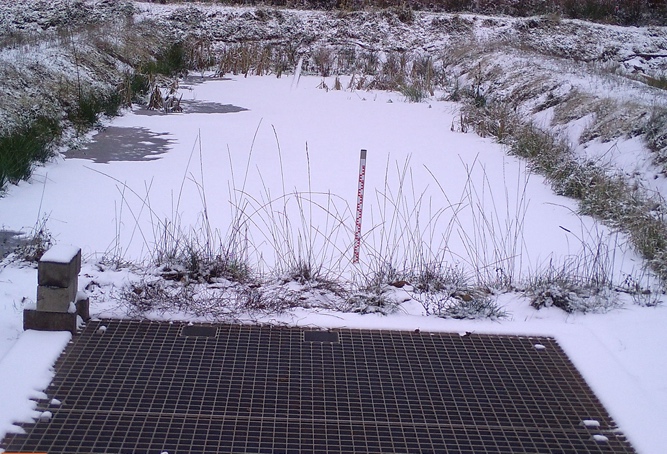

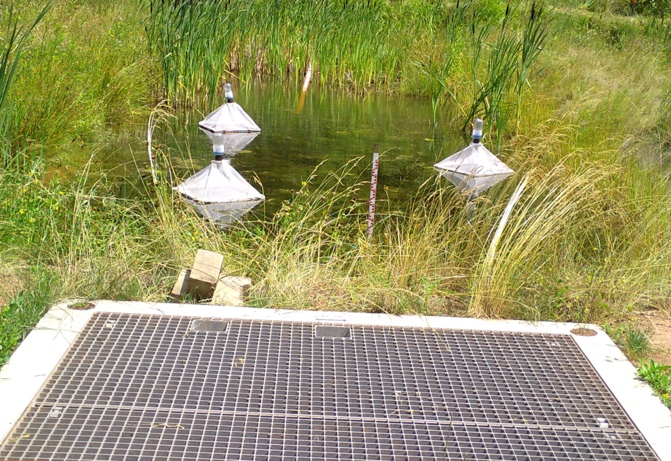


F

**Figure S2.** Exemplary photos of the ecosystem development of FPM10 from October 2017 till July 2019. Photos showing FPM10 in (A) October 2017, (B) November 2017, (C) March 2018, (D) August 2018, (E) January 2019, (F) July 2019. Please see also <https://youtu.be/vfTNpwyYhFE> for a time lapse video of the ecosystem development during the study period.

**Table S1.** Overview of the monitoring program, sampling locations, sampling methods and sampling intervals.

| **Parameter** | **Sampling location**^†^ | **Method (sampling interval)** |
| --- | --- | --- |
| ***General information on EERES site and FPM conditions*** | | |
| Ecosystem development of the floodplain mesocosms | FPM 1-12, banks and water-land floodplain area | Manual photos (weekly); automatic photos by camera trap (twice daily) |
| Weather data | Eußerthal / Siebeldingen | Meteorological station (Hourly) |
| Soil temperature (5 cm depth) | North and south banks of FPM 1-12 | Digital probe thermometer (monthly) |
| Water level | FPM 1 -12 | Level gauge (monthly) |
| ***Physico-chemical water quality parameters*** | |  |
| Water chemistry (DOC, TOC, anions, cations) | FPM 1-12 | Analytical measurement (monthly – bimonthly) |
| Specific conductivity | FPM 1-12 | Portable analytical device (monthly) |
| pH | FPM 1-12 | Portable analytical device (monthly) |
| Water temperature | FPM 1-12 | Temperature logger (10 minutes) |
| Dissolved oxygen | FPM 1-12 | O_2_ logger (10 minutes) |
| ***Biotic parameters*** |  |  |
| Benthic macroinvertebrates | FPM 1-12 | Three pebble baskets per FPM (monthly – bimonthly) |
| Merolimnic insect emergence | FPM 1-12 | Three emergence tents per FPM (weekly) |
| Zooplankton | FPM 1-12 | Plankton net (monthly) |
| Amphibians (tadpoles) | FPM 1-12 | Visual inspection of percentage tadpole coverage (daily – weekly while present) |
| Submerged vegetation | FPM 1-12 | Visual inspection of percentage filamentous algae and *Elodea* coverage (14 d – monthly) |
| Crayfish and fishes | FPM 1-12 | Three crayfish traps per FPM (monthly) |
| Terrestrial vegetation | Banks and water-land floodplain areas of FPM 1-12 | Plant mapping (yearly) |
| Ground beetles | Banks and water-land floodplain areas of FPM 1-12 | Four pitfall traps (twice in 2018 and once in 2019) |
| Leafhoppers | Banks of FPM3, FPM5, FPM8, FPM10 | Suction sampling (June and September 2018) |
| **Functional parameters** |  |  |
| Aquatic leaf litter decomposition | FPM 1-12 | Three coarse and fine leaf bags per FPM (monthly) |
| Terrestrial litter decomposition | North and south banks of FPM 1-12 | Two tea bags (rooibos and green tea) (monthly – every three months) |

†: If not specified otherwise, FPM 1-12 indicates that the sampling took place in the water body of all twelve FPMs.

**Supporting Results**

**Meteorology**

The meteorological data for the study period were derived from two weather stations in the vicinity (8 km and 1 km distance) to the EERES site. Rainfall data (mm per hour and per day) were taken from the meteorological station Eußerthal (<https://bit.ly/31XVF75>) and air temperature (C°, hourly and daily mean), as well as global radiation (Wh/m^2^, sum per day and month) from the meteorological station Siebeldingen (<https://bit.ly/2OxOTl9>). The meteorological data for the study period (06 November 2017 – 31 July 2019) are provided in Figure S3 and Table S2. The air temperature had minima in February 2018 (hourly minimum of -10.6 °C) and January 2019 (hourly minimum of -7.8 °C), whereas maximum temperatures were recorded at the end of July and beginning of August 2018 (hourly maximum of 34.3 °C), as well as in June and July 2019 (hourly maximum 36.8 °C) (Figure S3A; Table S2). The daily sum of global radiation shows minima below 100 Wh/m^2^ in the winter month of 2017/2018 and 2018/2019, whereas maxima of > 8000 Wh/m^2^ occurred in June and July of 2018 and 2019 (Figure S3B); highest sum global radiations per month were recorded for July 2018 and July 2019 (Table S2). The precipitation data revealed particularly dry periods in July and October 2018, with precipitation sums of less than 20 mm per month (Figure S3C; Table S2). In contrast, high precipitation amounts occurred at the beginning of the study period (i.e., November 2017 – January 2018), as well as in May 2018, in which also the highest amount of rainfall per hour (40.23 mm) occurred (Figure S3C; Table S2).

**Soil temperature**

The soil temperature in 5 cm depth was measured monthly on the top of the northern and southern banks of each FPM using a digital probe thermometer (TFA Dostmann). The soil temperature followed a seasonal pattern with lowest temperatures in the winter months and highest during summer (Figure S4). The soil temperatures were generally higher at the northern banks due to higher solar irradiation reasoned by their southward direction (Figure S4; Table S3). Differences in soil temperature between individual ponds occurred particularly during the summer months (Figure S4); these differences are, however, likely the results of measurement bias, i.e., the temperature was in parts measured at different times of the day at the individual ponds. However, the median values of the soil temperature of both the northern and southern banks were comparable indicating no general differences between FPMs (Table S3).

**Water level**

The water levels of the twelve FPMs were recorded monthly from a level gauge at the outlet site of the ponds. Overall, the levels were comparable across the twelve ponds, with median water levels of 30 cm documented for all FPMs (Table S4). Individual outlier values of 20 cm (FPM11) and 38 cm (FPM1) in February and March 2018 are related to pond icing. However, no general increasing or decreasing trend exists for the water levels over the study period (Figure S5).


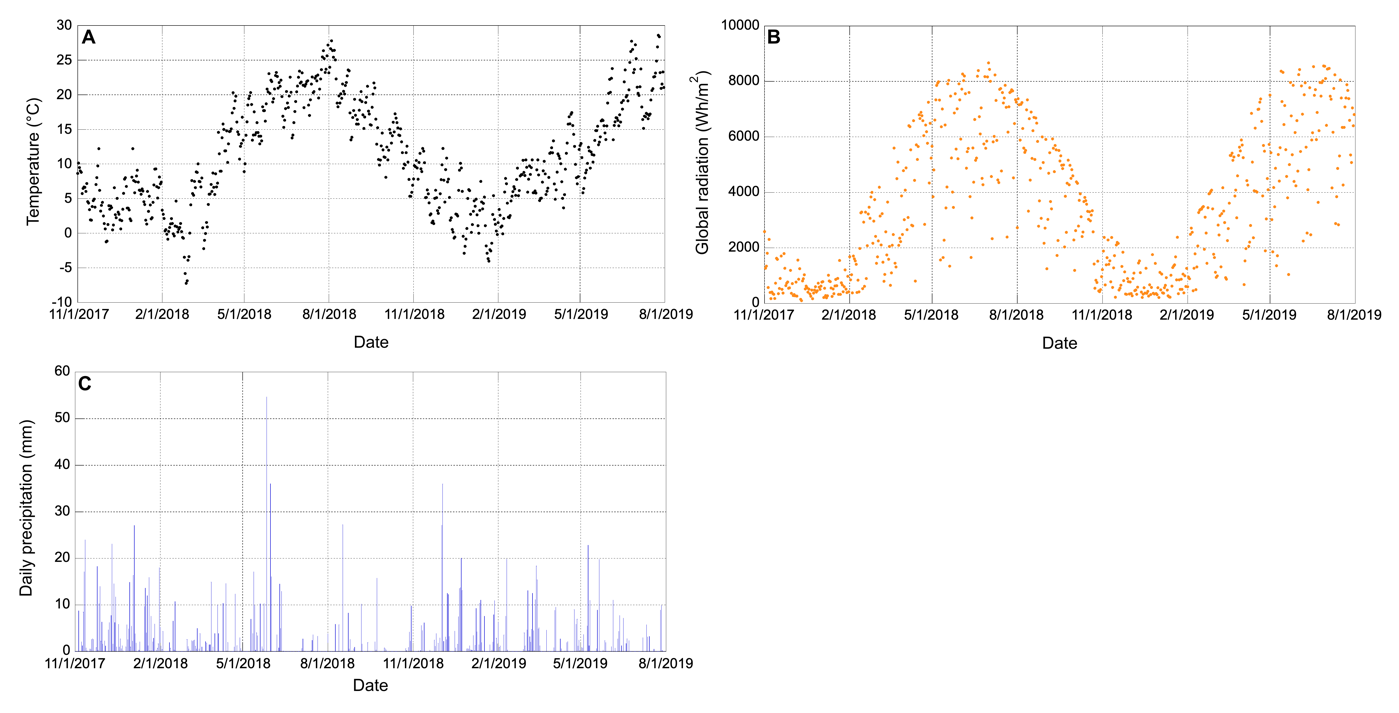


**Figure S3.** Meteorological data for the EERES site, with (A) air temperature in °C (daily mean), (B) global radiation in Wh/m^2^ (daily sum per day) and (C) precipitation in mm (sum per day) displayed for the study period 06 November 2017 to 31 July 2019.

**Figure S4.** Soil temperature in 5 cm depth of (A) southern and (B) northern banks of the twelve FPMs. Black circles depict means and error bars show 95% confidence intervals.

**Figure S5.** Water levels of FPM 1-12 recorded from level gauges during the study period. Black circles depict means and error bars show 95% confidence intervals.

**Table S2.** Monthly characteristics of precipitation amounts, air temperature and global radiation for the EERES site.

| **Date** | **Precipitation (mm) per hour** | | | | | **Air temperature (°C)** | | | | **Global radiation (Wh/m^2^)** |
| --- | --- | --- | --- | --- | --- | --- | --- | --- | --- | --- |
|  | **Mean** | **Median** | **Max.** | **Min.** | **Sum per month** | **Mean** | **Median** | **Max.** | **Min.** | **Sum per month** |
| Nov 17 | 0.17 | 0.00 | 6.78 | 0.00 | 123.6 | 5.90 | 5.70 | 17.30 | -1.00 | 27892 |
| Dec 17 | 0.17 | 0.00 | 4.00 | 0.00 | 123.1 | 3.53 | 2.90 | 14.10 | -2.50 | 17273 |
| Jan 18 | 0.21 | 0.00 | 7.06 | 0.00 | 154.7 | 5.87 | 6.20 | 12.10 | -2.80 | 19122 |
| Feb 18 | 0.05 | 0.00 | 2.19 | 0.00 | 31.5 | -0.05 | 0.50 | 9.50 | -10.60 | 57171 |
| Mar 18 | 0.07 | 0.00 | 3.28 | 0.00 | 48.9 | 4.68 | 5.55 | 13.50 | -7.50 | 77496 |
| Apr 18 | 0.09 | 0.00 | 9.61 | 0.00 | 63.1 | 13.83 | 13.30 | 27.10 | -0.30 | 154311 |
| May 18 | 0.21 | 0.00 | 40.23 | 0.00 | 158.4 | 16.83 | 16.40 | 29.10 | 2.20 | 177209 |
| Jun 18 | 0.07 | 0.00 | 14.45 | 0.00 | 52.9 | 19.55 | 19.10 | 30.10 | 7.60 | 183302 |
| Jul 18 | 0.02 | 0.00 | 2.00 | 0.00 | 12.9 | 22.16 | 21.25 | 34.30 | 12.90 | 202881 |
| Aug 18 | 0.08 | 0.00 | 23.06 | 0.00 | 55.8 | 20.95 | 20.20 | 34.20 | 6.80 | 168742 |
| Sep 18 | 0.04 | 0.00 | 11.70 | 0.00 | 31.8 | 16.35 | 16.10 | 30.40 | 2.60 | 130776 |
| Oct 18 | 0.03 | 0.00 | 2.51 | 0.00 | 19.6 | 11.93 | 11.50 | 25.70 | 0.70 | 77223 |
| Nov 18 | 0.04 | 0.00 | 2.93 | 0.00 | 29.6 | 6.60 | 5.90 | 16.70 | -2.40 | 37330 |
| Dec 18 | 0.24 | 0.00 | 5.39 | 0.00 | 175.1 | 4.54 | 5.00 | 13.10 | -4.50 | 20264 |
| Jan 19 | 0.11 | 0.00 | 2.60 | 0.00 | 80.1 | 1.70 | 1.90 | 8.80 | -7.80 | 24924 |
| Feb 19 | 0.06 | 0.00 | 3.64 | 0.00 | 40.1 | 5.11 | 4.50 | 18.00 | -3.40 | 69869 |
| Mar 19 | 0.15 | 0.00 | 3.77 | 0.00 | 110.8 | 8.16 | 8.15 | 19.90 | -1.20 | 104757 |
| Apr 19 | 0.07 | 0.00 | 7.12 | 0.00 | 52.0 | 10.73 | 10.10 | 23.80 | -0.20 | 133114 |
| May 19 | 0.11 | 0.00 | 5.67 | 0.00 | 79.6 | 12.53 | 12.30 | 23.60 | 1.60 | 160382 |
| Jun 19 | 0.06 | 0.00 | 7.42 | 0.00 | 42.7 | 20.42 | 19.70 | 36.50 | 8.30 | 196863 |
| Jul 19 | 0.05 | 0.00 | 8.41 | 0.00 | 34.5 | 20.91 | 20.20 | 36.80 | 8.30 | 199729 |

**Table S3.** Statistical characteristics of the soil temperature (°C) at 5 cm depth of the southern and northern FPM banks.

| **South bank** | **FPM1** | **FPM2** | **FPM3** | **FPM4** | **FPM5** | **FPM6** | **FPM7** | **FPM8** | **FPM9** | **FPM10** | **FPM11** | **FPM12** |
| --- | --- | --- | --- | --- | --- | --- | --- | --- | --- | --- | --- | --- |
| Minimum | -3.0 | -3.0 | -3.0 | -2.0 | -3.0 | -2.0 | -2.0 | -3.0 | -2.0 | -3.0 | -3.0 | -1.0 |
| 25th percentile | 3.8 | 4.5 | 4.3 | 4.0 | 4.0 | 4.3 | 4.3 | 4.3 | 4.5 | 4.3 | 4.0 | 4.5 |
| Median | 6.0 | 7.0 | 8.0 | 8.0 | 8.0 | 7.8 | 6.8 | 6.8 | 6.2 | 8.0 | 8.0 | 8.0 |
| 75th percentile | 11.8 | 15.6 | 14.0 | 15.0 | 16.4 | 12.1 | 13.5 | 11.6 | 12.5 | 11.5 | 9.8 | 12.5 |
| 90th percentile | 25.2 | 22.4 | 23.0 | 25.4 | 24.4 | 21.4 | 21.2 | 20.0 | 20.0 | 21.0 | 18.2 | 18.3 |
| Maximum | 27.0 | 27.5 | 30.0 | 45.0 | 28.5 | 24.0 | 23.0 | 22.0 | 21.0 | 21.5 | 20.0 | 20.0 |
| Mean | 9.6 | 10.1 | 10.3 | 11.5 | 10.6 | 9.6 | 9.5 | 9.0 | 9.0 | 8.9 | 8.2 | 8.8 |
| **North bank** | **FPM1** | **FPM2** | **FPM3** | **FPM4** | **FPM5** | **FPM6** | **FPM7** | **FPM8** | **FPM9** | **FPM10** | **FPM11** | **FPM12** |
| Minimum | 1.0 | 0.0 | 1.0 | 1.5 | 1.1 | 1.3 | 1.0 | 0.0 | 0.5 | 0.8 | 0.0 | 0.5 |
| 25th percentile | 5.3 | 5.4 | 4.7 | 5.0 | 5.0 | 5.0 | 5.0 | 5.0 | 4.8 | 4.8 | 5.0 | 4.6 |
| Median | 10.0 | 9.0 | 9.0 | 8.3 | 9.0 | 10.0 | 8.5 | 8.5 | 10.0 | 8.0 | 7.5 | 9.5 |
| 75th percentile | 21.4 | 19.0 | 19.0 | 19.8 | 22.0 | 18.5 | 17.5 | 15.0 | 17.0 | 13.3 | 12.0 | 17.5 |
| 90th percentile | 31.2 | 29.2 | 28.2 | 29.0 | 31.2 | 28.7 | 25.3 | 24.1 | 23.0 | 20.2 | 19.8 | 21.8 |
| Maximum | 35.0 | 32.0 | 29.0 | 35.0 | 36.0 | 38.0 | 32.0 | 29.0 | 26.0 | 21.0 | 24.5 | 25.0 |
| Mean | 14.0 | 12.3 | 12.3 | 12.7 | 13.8 | 13.1 | 11.3 | 11.4 | 11.2 | 9.8 | 9.2 | 11.2 |

**Table S4.** Statistical characteristics of the water levels of the FPMs during study period (06 November 2017 – 31 July 2019).

| **Water level (cm)** | **FPM1** | **FPM2** | **FPM3** | **FPM4** | **FPM5** | **FPM6** | **FPM7** | **FPM8** | **FPM9** | **FPM10** | **FPM11** | **FPM12** |
| --- | --- | --- | --- | --- | --- | --- | --- | --- | --- | --- | --- | --- |
| Minimum | 25.0 | 28.0 | 27.0 | 27.5 | 28.0 | 27.5 | 28.0 | 29.5 | 28.5 | 29.0 | 20.0 | 28.0 |
| 25th percentile | 29.0 | 29.5 | 28.8 | 29.0 | 30.0 | 29.0 | 30.0 | 30.0 | 29.0 | 30.0 | 29.0 | 30.0 |
| Median | 30.0 | 30.0 | 30.0 | 30.0 | 30.0 | 30.0 | 30.0 | 30.0 | 30.0 | 30.0 | 30.0 | 30.0 |
| 75th percentile | 30.0 | 30.3 | 30.0 | 30.0 | 31.0 | 30.0 | 31.0 | 30.0 | 30.0 | 31.0 | 30.4 | 30.0 |
| 90th percentile | 31.0 | 31.0 | 31.0 | 31.2 | 31.0 | 31.0 | 31.2 | 31.0 | 30.3 | 31.0 | 31.0 | 31.2 |
| Maximum | 38.0 | 31.0 | 33.0 | 33.0 | 32.0 | 32.0 | 32.0 | 32.0 | 32.0 | 32.0 | 33.0 | 31.2 |
| Mean | 29.7 | 29.8 | 29.5 | 29.8 | 30.1 | 29.6 | 30.3 | 30.2 | 29.7 | 30.4 | 28.8 | 30.1 |

**Physico-chemical water quality parameters**

Concentration levels of the inorganic ions and organic carbon contents differed between the individual components, with highest concentrations measured for calcium and lowest for nitrite (Table S5). Across the study period, TOC, DOC, chloride, sulfate and calcium concentrations showed tendencies of seasonal patterns, with slight maxima in summer and early autumn 2018 and lower concentrations during winter 2018/2019 (Figure S6). However, concentrations of fluoride, nitrate and potassium tend to decrease over the study period, whereas phosphate concentrations showed general increasing trends from November 2018 onwards. The concentrations of sodium and magnesium were rather constant during the study period; concentrations of ammonium and nitrite were mostly below the limit of quantification (LOQ), with, however, distinct increased levels particularly during summer and autumn 2018 (Figure S6). Importantly, the concentrations of most components showed a rather high inter-pond variability at the individual sampling dates. However, no pond showed generally higher or lower concentrations across the study period when considering all water chemistry components (Table S5).

The specific conductivity averaged across all ponds was generally between 100 and 200 µS/cm during the study period (Figure S7A). The conductivity increased from low levels in winter 2017/2018 (mean conductivity across all ponds of 97.8 µS/cm; 95% CI [95.5, 100.2]) to a maximum value at the beginning of May 2018 (mean of 266 µS/cm; 95% CI [255.1, 277.1]) and subsequently decreased to lower values in autumn and winter 2018. Subsequent to lower specific conductivities in May 2019 (mean of 101 µS/cm; 95% CI [83.9, 117.9]), an increase till the end of July 2019 was observed (Figure S7A). In November 2017 practically no variation existed between the ponds. However, the variation between the FPMs increased over time with differences of >200 µS/cm at the end of the study period (Figure S7A). Based on mean and median values (Table S6), FPM8, FPM9, FPM10, FPM12 had lower specific conductivities (median </= 120 µS/cm), whereas FPM1, FPM3, FPM4, and FPM7 were characterized by higher specific conductivities (mean and median > 150 µS/cm). However, apart from FPM4 with a maximum of 330 µS/cm, specific conductivity did not exceed 300 µS/cm in the other ponds (Figure S7A; Table S6).

The pH values averaged across the twelve FPMs were mostly in the range of 7.5 to 9.5 during the entire study period (Figure S7B). Despite some variations, the pH values showed an generally increasing trend from November 2017 (mean pH across all ponds of 7.9; 95% CI [7.7, 8.1]) to a maximum in September 2018 (mean pH of 9.6; 95% CI [9.4, 9.9]). In 2019, average pH values were rather constant between 8.5 and 9 but decreased in July 2019 to values between 7.5 and 8 (Figure S7B). The individual pH values of the FPMs were generally in the range between 7.2 and 10 during the study period (Figure S7B). However, a rather large variation (i.e., up to more than 2.5 pH unit difference) was recorded between individual FPMs for individual sampling dates, particularly from April 2019 onwards. Based on mean and median pH values of the individual ponds, slightly higher pH values were recorded in FPM2, FPM5 and FPM 8-12 (median and mean pH >/= 8.5) compared with the other ponds (Table S6).

The water temperature in 25 cm depth showed a clear seasonal pattern, with lowest temperatures in winter months and highest temperatures during summer (Figure S7C). Temperatures did not fall below 2°C and the maximum temperature did not exceed 26.1°C (Table S6). The water temperature did not differ largely between the individual FPMs (Figure S7C). However, care has to be taken when comparing the temperatures of the FPM9-FPM12 due to missing values for individual months (see Table S6).

The dissolved oxygen concentrations increased from the beginning of November till end of December 2017 and dropped from March 2018 to low oxygen concentrations in spring and summer 2018 (Figure S7D); a subsequent increase of dissolved oxygen concentrations till February 2019 was followed by a decrease till the end of July 2019. The dissolved oxygen concentrations showed thus an opposing trend to water temperature, with higher dissolved oxygen concentrations in winter months and lower during summer months. The variation of dissolved oxygen concentrations between the individual FPMs increased over time with particularly high variations (i.e., differences of up to >25 mg/L) occurring in winter 2018/2019 (Figure S7D). Excepting FPM8, which had generally higher dissolved oxygen concentrations, no large differences exist between the median and mean oxygen concentrations of the FPMs (Table S6). Maxima of dissolved oxygen concentrations were generally <20 mg/L, with, however, maximum concentrations of >20 mg/l (up to 26.8 mg/L) were recorded for FPM2, FPM8, and FPM 10-12 (Figure S7D; Table S6). Again, care have to be taken in interpreting the oxygen concentrations of individual FPMs due to logger failures particularly for FPM 9-12 (Table S6).

**Figure S6.** Concentrations of (A) TOC, (B) DOC, (C) fluoride, (D) chloride, (E) nitrite, (F) nitrate, (G) phosphate, (H) sulfate, (I) sodium, (J) ammonium, (K) potassium, (L) calcium, and (M) magnesium in the twelve FPMs over time (6 November 2017 – 31 July 2019). Black circles depict means and error bars show 95% confidence intervals.

**Figure S7**. Development of (A) specific conductivity, (B) pH-value, (C) water temperature, and (D) dissolved oxygen concentration for FPM 1-12 over time (6 November 2017 – 31 July 2019). Black circles depict means and error bars show 95% confidence intervals. Missing values are due to equipment failure (see Table S6).

**Table S5.** Statistical characteristics of the water chemistry (i.e., concentrations of anions, cations, total organic carbon (TOC), dissolved organic carbon (DOC)) for the study period (06 November 2017 – 31 July 2019). Concentrations less than the limit of quantification (LOQ) were replaced by 0.5 x LOQ.

| **TOC (mg/L)** | **FPM1** | **FPM2** | **FPM3** | **FPM4** | **FPM5** | **FPM6** | **FPM7** | **FPM8** | **FPM9** | **FPM10** | **FPM11** | **FPM12** |
| --- | --- | --- | --- | --- | --- | --- | --- | --- | --- | --- | --- | --- |
| Minimum | 3.0000 | 3.1000 | 3.4201 | 2.4263 | 2.9000 | 3.1832 | 2.8000 | 2.4000 | 3.1000 | 2.2962 | 3.3000 | 2.9805 |
| 25th percentile | 4.0500 | 3.4244 | 4.0000 | 3.6500 | 3.1500 | 3.8500 | 3.6000 | 3.2998 | 3.4000 | 3.4500 | 3.5500 | 3.5500 |
| Median | 4.7000 | 4.0000 | 4.6000 | 4.2363 | 3.4000 | 4.4000 | 4.2000 | 3.9000 | 4.1000 | 4.4000 | 4.7837 | 4.2000 |
| Mean | 5.1253 | 4.3792 | 5.3048 | 4.5581 | 4.1331 | 4.7738 | 4.8498 | 3.9166 | 4.5060 | 5.4022 | 5.1307 | 5.5796 |
| 75th percentile | 6.4308 | 5.1252 | 5.8000 | 5.1956 | 4.5500 | 5.3500 | 5.1401 | 4.4495 | 5.3500 | 5.8500 | 5.6029 | 6.6000 |
| 90th percentile | 6.7000 | 5.5000 | 7.2000 | 5.6100 | 5.6426 | 5.7000 | 5.8000 | 5.0000 | 6.3440 | 9.8919 | 7.0000 | 9.9154 |
| Maximum | 6.7837 | 7.2720 | 10.486 | 8.4000 | 8.1830 | 8.3899 | 10.638 | 5.6845 | 6.9584 | 12.136 | 11.148 | 11.380 |
| **DOC (mg/L)** | **FPM1** | **FPM2** | **FPM3** | **FPM4** | **FPM5** | **FPM6** | **FPM7** | **FPM8** | **FPM9** | **FPM10** | **FPM11** | **FPM12** |
| Minimum | 2.6106 | 2.3183 | 2.4602 | 2.1623 | 2.3270 | 2.2302 | 2.4564 | 2.2737 | 2.3888 | 2.4585 | 2.5409 | 2.9405 |
| 25th percentile | 3.9000 | 3.5000 | 3.8827 | 3.2996 | 3.1000 | 3.6430 | 3.7500 | 3.2779 | 3.3000 | 3.7559 | 3.4500 | 3.4038 |
| Median | 4.7000 | 4.1622 | 4.4000 | 3.9309 | 3.4914 | 4.1783 | 4.1672 | 3.8920 | 4.0000 | 4.1590 | 3.7670 | 4.1898 |
| Mean | 5.1137 | 4.3169 | 4.6641 | 4.1049 | 4.0766 | 4.6011 | 4.7769 | 3.8994 | 4.2071 | 5.5283 | 4.3278 | 4.8301 |
| 75th percentile | 6.1000 | 4.5318 | 5.0434 | 4.3832 | 4.5267 | 4.9000 | 5.0500 | 4.4695 | 4.7776 | 5.6853 | 4.9815 | 5.9041 |
| 90th percentile | 7.2727 | 5.5200 | 5.6400 | 5.4023 | 5.4218 | 6.7328 | 7.4327 | 5.4811 | 5.8366 | 10.484 | 5.4481 | 7.1600 |
| Maximum | 8.9608 | 8.4478 | 9.1169 | 8.1000 | 8.9780 | 9.2000 | 10.000 | 5.8000 | 7.6200 | 13.200 | 8.5700 | 9.3100 |
| **Fluoride (mg/L)** | **FPM1** | **FPM2** | **FPM3** | **FPM4** | **FPM5** | **FPM6** | **FPM7** | **FPM8** | **FPM9** | **FPM10** | **FPM11** | **FPM12** |
| Minimum | 0.0250 | 0.0250 | 0.0250 | 0.0250 | 0.0250 | 0.0250 | 0.0250 | 0.0250 | 0.0250 | 0.0728 | 0.0600 | 0.0800 |
| 25th percentile | 0.0800 | 0.0700 | 0.0650 | 0.0313 | 0.0613 | 0.0725 | 0.0800 | 0.0293 | 0.0675 | 0.0950 | 0.1000 | 0.1000 |
| Median | 0.0900 | 0.0863 | 0.0800 | 0.0625 | 0.0818 | 0.0950 | 0.0935 | 0.0600 | 0.0900 | 0.1100 | 0.1200 | 0.1091 |
| Mean | 0.0959 | 0.0820 | 0.0862 | 0.0641 | 0.0808 | 0.0928 | 0.0933 | 0.0566 | 0.0854 | 0.1171 | 0.1278 | 0.1140 |
| 75th percentile | 0.1300 | 0.1004 | 0.1134 | 0.0928 | 0.1077 | 0.1175 | 0.1134 | 0.0798 | 0.0975 | 0.1129 | 0.1473 | 0.1300 |
| 90th percentile | 0.1371 | 0.1259 | 0.1299 | 0.1073 | 0.1137 | 0.1218 | 0.1270 | 0.0844 | 0.1152 | 0.1859 | 0.1749 | 0.1483 |
| Maximum | 0.1444 | 0.1299 | 0.1444 | 0.1153 | 0.1195 | 0.1517 | 0.1300 | 0.0890 | 0.1444 | 0.2170 | 0.2170 | 0.1500 |
| **Chloride (mg/L)** | **FPM1** | **FPM2** | **FPM3** | **FPM4** | **FPM5** | **FPM6** | **FPM7** | **FPM8** | **FPM9** | **FPM10** | **FPM11** | **FPM12** |
| Minimum | 0.5000 | 0.5000 | 0.5000 | 0.5000 | 1.2000 | 0.5000 | 0.5000 | 0.5000 | 0.5000 | 0.5000 | 0.5000 | 0.5000 |
| 25th percentile | 1.2945 | 1.1793 | 1.1890 | 1.2644 | 1.7000 | 1.2000 | 1.2979 | 1.6000 | 1.4013 | 0.6500 | 1.2867 | 1.2593 |
| Median | 1.6000 | 1.5986 | 1.3023 | 1.4143 | 1.9965 | 1.4289 | 1.6995 | 2.1000 | 1.6699 | 1.3112 | 1.6081 | 1.6000 |
| Mean | 1.6822 | 1.5738 | 1.7396 | 1.5994 | 2.0354 | 1.5540 | 1.7731 | 2.1131 | 1.8665 | 1.6818 | 1.8816 | 1.7908 |
| 75th percentile | 2.0897 | 1.9617 | 2.1127 | 1.8000 | 2.1962 | 1.9775 | 1.9706 | 2.7000 | 2.4763 | 2.5250 | 2.1750 | 2.5000 |
| 90th percentile | 2.8671 | 2.2000 | 2.9716 | 2.2800 | 3.0338 | 2.7553 | 2.9295 | 3.0549 | 2.6515 | 3.1717 | 3.3047 | 2.9794 |
| Maximum | 3.0093 | 2.9000 | 4.2000 | 3.4469 | 3.4000 | 3.0214 | 3.2000 | 3.3000 | 3.1430 | 3.3561 | 4.2000 | 3.2135 |
| **Nitrite (mg/L)** | **FPM1** | **FPM2** | **FPM3** | **FPM4** | **FPM5** | **FPM6** | **FPM7** | **FPM8** | **FPM9** | **FPM10** | **FPM11** | **FPM12** |
| Minimum | 0.0005 | 0.0005 | 0.0002 | 0.0001 | 0.0001 | 0.0001 | 0.0004 | 0.0001 | 0.0005 | 0.0004 | 0.0005 | 0.0005 |
| 25th percentile | 0.0005 | 0.0005 | 0.0005 | 0.0005 | 0.0005 | 0.0005 | 0.0005 | 0.0005 | 0.0005 | 0.0005 | 0.0005 | 0.0005 |
| Median | 0.0005 | 0.0005 | 0.0005 | 0.0005 | 0.0005 | 0.0005 | 0.0005 | 0.0005 | 0.0005 | 0.0005 | 0.0005 | 0.0005 |
| Mean | 0.0019 | 0.0016 | 0.0014 | 0.0008 | 0.0012 | 0.0022 | 0.0018 | 0.0016 | 0.0016 | 0.0017 | 0.0022 | 0.0014 |
| 75th percentile | 0.0005 | 0.0005 | 0.0005 | 0.0005 | 0.0005 | 0.0005 | 0.0005 | 0.0005 | 0.0007 | 0.0005 | 0.0007 | 0.0005 |
| 90th percentile | 0.0066 | 0.0038 | 0.0038 | 0.0005 | 0.0038 | 0.0066 | 0.0038 | 0.0060 | 0.0056 | 0.0060 | 0.0062 | 0.0038 |
| Maximum | 0.0090 | 0.0110 | 0.0090 | 0.0050 | 0.0060 | 0.0140 | 0.0150 | 0.0060 | 0.0070 | 0.0070 | 0.0140 | 0.0080 |
| **Nitrate (mg/L)** | **FPM1** | **FPM2** | **FPM3** | **FPM4** | **FPM5** | **FPM6** | **FPM7** | **FPM8** | **FPM9** | **FPM10** | **FPM11** | **FPM12** |
| Minimum | 0.0500 | 0.0500 | 0.0500 | 0.0500 | 0.0500 | 0.0500 | 0.0500 | 0.0500 | 0.0500 | 0.0500 | 0.0500 | 0.0500 |
| 25th percentile | 0.0500 | 0.0500 | 0.0500 | 0.0500 | 0.0500 | 0.0500 | 0.0500 | 0.0500 | 0.0500 | 0.0500 | 0.0500 | 0.0500 |
| Median | 0.0500 | 0.0500 | 0.0500 | 0.0500 | 0.0500 | 0.0883 | 0.0500 | 0.0500 | 0.0500 | 0.0500 | 0.0500 | 0.0500 |
| Mean | 0.2509 | 0.2468 | 0.2372 | 0.2097 | 0.2519 | 0.4107 | 0.3094 | 0.2636 | 0.3280 | 0.4341 | 0.3786 | 0.4076 |
| 75th percentile | 0.1007 | 0.2386 | 0.1007 | 0.0500 | 0.0500 | 0.4861 | 0.0500 | 0.0500 | 0.1944 | 0.1515 | 0.1132 | 0.1448 |
| 90th percentile | 0.7237 | 0.8025 | 0.6839 | 0.2899 | 0.8151 | 1.4113 | 1.1441 | 0.6331 | 1.0835 | 1.1060 | 1.5713 | 1.6846 |
| Maximum | 1.7633 | 1.2143 | 1.5724 | 1.7872 | 1.6201 | 1.8578 | 2.0503 | 2.1214 | 1.8827 | 3.7252 | 1.9541 | 2.0975 |
| **Phosphate (mg/L)** | **FPM1** | **FPM2** | **FPM3** | **FPM4** | **FPM5** | **FPM6** | **FPM7** | **FPM8** | **FPM9** | **FPM10** | **FPM11** | **FPM12** |
| Minimum | 0.0250 | 0.0250 | 0.0250 | 0.0250 | 0.0250 | 0.0250 | 0.0250 | 0.0250 | 0.0250 | 0.0250 | 0.0250 | 0.0200 |
| 25th percentile | 0.0250 | 0.0250 | 0.0250 | 0.0250 | 0.0250 | 0.0250 | 0.0250 | 0.0250 | 0.0250 | 0.0250 | 0.0250 | 0.0250 |
| Median | 0.0250 | 0.0250 | 0.0250 | 0.0250 | 0.0250 | 0.0250 | 0.0250 | 0.0250 | 0.0250 | 0.0250 | 0.0250 | 0.0250 |
| Mean | 0.0486 | 0.0354 | 0.0468 | 0.0471 | 0.0404 | 0.0458 | 0.0377 | 0.0358 | 0.0311 | 0.0443 | 0.0368 | 0.0504 |
| 75th percentile | 0.0700 | 0.0250 | 0.0675 | 0.0725 | 0.0500 | 0.0600 | 0.0250 | 0.0250 | 0.0250 | 0.0588 | 0.0250 | 0.0800 |
| 90th percentile | 0.0840 | 0.0640 | 0.0840 | 0.0800 | 0.0780 | 0.1040 | 0.0660 | 0.0770 | 0.0250 | 0.0870 | 0.0710 | 0.0980 |
| Maximum | 0.1200 | 0.1000 | 0.1400 | 0.1100 | 0.1000 | 0.1200 | 0.1200 | 0.1000 | 0.1100 | 0.1300 | 0.1100 | 0.1400 |
| **Sulfate (mg/L)** | **FPM1** | **FPM2** | **FPM3** | **FPM4** | **FPM5** | **FPM6** | **FPM7** | **FPM8** | **FPM9** | **FPM10** | **FPM11** | **FPM12** |
| Minimum | 0.5000 | 0.5000 | 1.1000 | 0.5000 | 0.5000 | 1.2000 | 0.5000 | 0.5000 | 0.5000 | 0.5000 | 0.5000 | 0.5000 |
| 25th percentile | 2.3106 | 4.2473 | 5.0077 | 2.8609 | 2.7805 | 4.4000 | 3.6444 | 2.8000 | 2.3250 | 1.1250 | 3.1273 | 1.4000 |
| Median | 5.5311 | 6.2883 | 6.8301 | 3.9655 | 4.4105 | 6.3000 | 5.5880 | 5.6000 | 4.0457 | 3.3058 | 5.4298 | 5.4501 |
| Mean | 4.8858 | 5.4993 | 6.5686 | 4.2477 | 4.5303 | 6.0042 | 5.2774 | 5.4106 | 4.3215 | 3.6518 | 6.0352 | 4.3439 |
| 75th percentile | 6.6774 | 7.3750 | 8.0500 | 5.7545 | 5.0000 | 7.5000 | 7.3000 | 8.1910 | 6.5250 | 6.0250 | 8.3354 | 6.3089 |
| 90th percentile | 7.9312 | 7.5400 | 8.7953 | 6.8100 | 7.9763 | 8.7295 | 9.4531 | 8.5983 | 7.8319 | 7.7308 | 11.410 | 7.0000 |
| Maximum | 10.927 | 9.5000 | 11.649 | 9.8214 | 10.100 | 11.000 | 10.100 | 10.500 | 8.5000 | 8.6954 | 13.500 | 8.3842 |
| **Sodium (mg/L)** | **FPM1** | **FPM2** | **FPM3** | **FPM4** | **FPM5** | **FPM6** | **FPM7** | **FPM8** | **FPM9** | **FPM10** | **FPM11** | **FPM12** |
| Minimum | 0.9060 | 1.2300 | 1.1200 | 0.8890 | 0.6306 | 0.9495 | 0.9039 | 1.0300 | 0.6864 | 0.5520 | 1.1300 | 0.3120 |
| 25th percentile | 1.2750 | 1.6800 | 1.7800 | 1.2549 | 1.3550 | 1.2870 | 1.4550 | 1.2404 | 1.3750 | 1.4000 | 1.2450 | 1.1200 |
| Median | 1.4900 | 1.8780 | 2.2204 | 1.5303 | 1.8900 | 1.4600 | 1.6960 | 1.8459 | 1.5400 | 1.7922 | 1.8036 | 1.6300 |
| Mean | 1.7687 | 2.2412 | 2.1323 | 1.5455 | 1.9731 | 1.7864 | 1.7041 | 1.8027 | 1.6006 | 2.1117 | 2.0077 | 1.6876 |
| 75th percentile | 1.9677 | 2.1062 | 2.4950 | 1.8322 | 2.2207 | 2.0710 | 2.0064 | 2.0493 | 1.9350 | 2.3184 | 2.1284 | 2.3075 |
| 90th percentile | 2.5225 | 3.8988 | 2.5719 | 2.0570 | 2.6053 | 2.4277 | 2.1674 | 2.1531 | 2.1818 | 2.9274 | 2.6618 | 2.8023 |
| Maximum | 4.2879 | 4.9805 | 3.1546 | 2.3234 | 5.2796 | 4.2124 | 2.2635 | 4.1116 | 2.5733 | 6.4609 | 6.0761 | 3.2058 |
| **Ammonium (mg/L)** | **FPM1** | **FPM2** | **FPM3** | **FPM4** | **FPM5** | **FPM6** | **FPM7** | **FPM8** | **FPM9** | **FPM10** | **FPM11** | **FPM12** |
| Minimum | 0.0100 | 0.0100 | 0.0100 | 0.0100 | 0.0100 | 0.0100 | 0.0100 | 0.0100 | 0.0100 | 0.0100 | 0.0100 | 0.0100 |
| 25th percentile | 0.0100 | 0.0100 | 0.0100 | 0.0100 | 0.0100 | 0.0100 | 0.0100 | 0.0100 | 0.0100 | 0.0100 | 0.0100 | 0.0100 |
| Median | 0.0100 | 0.0100 | 0.0100 | 0.0100 | 0.0100 | 0.0100 | 0.0100 | 0.0100 | 0.0100 | 0.0100 | 0.0100 | 0.0100 |
| Mean | 0.0252 | 0.0306 | 0.1544 | 0.0319 | 0.0473 | 0.0512 | 0.0434 | 0.0303 | 0.0652 | 0.1309 | 0.0347 | 0.1243 |
| 75th percentile | 0.0178 | 0.0100 | 0.0503 | 0.0175 | 0.0138 | 0.0235 | 0.0185 | 0.0100 | 0.0185 | 0.0765 | 0.0100 | 0.1107 |
| 90th percentile | 0.0495 | 0.0899 | 0.3111 | 0.0972 | 0.1600 | 0.1505 | 0.1430 | 0.0885 | 0.1675 | 0.2383 | 0.0974 | 0.2050 |
| Maximum | 0.1435 | 0.1800 | 1.6000 | 0.1702 | 0.2912 | 0.3579 | 0.2448 | 0.1782 | 0.5445 | 1.2382 | 0.2300 | 1.2027 |
| **Potassium (mg/L)** | **FPM1** | **FPM2** | **FPM3** | **FPM4** | **FPM5** | **FPM6** | **FPM7** | **FPM8** | **FPM9** | **FPM10** | **FPM11** | **FPM12** |
| Minimum | 0.0500 | 0.0500 | 1.3600 | 0.0500 | 0.0500 | 0.3940 | 0.2090 | 0.0500 | 0.0500 | 0.0500 | 0.0500 | 0.0500 |
| 25th percentile | 0.7880 | 0.9863 | 2.1344 | 0.2163 | 0.2450 | 1.4775 | 1.3375 | 0.2975 | 1.2075 | 1.1575 | 0.1965 | 0.4790 |
| Median | 2.0253 | 2.0012 | 2.5042 | 1.7782 | 2.1482 | 2.0473 | 1.7658 | 1.9531 | 1.8902 | 2.0246 | 2.0554 | 1.3600 |
| Mean | 1.9666 | 1.9292 | 2.7135 | 1.6986 | 1.6663 | 2.1317 | 1.8339 | 1.5500 | 1.9634 | 2.0022 | 1.7376 | 1.5319 |
| 75th percentile | 2.8378 | 2.4650 | 3.4464 | 2.7484 | 2.6247 | 2.6100 | 2.3956 | 2.4260 | 2.6423 | 2.6627 | 2.6819 | 2.5851 |
| 90th percentile | 3.4486 | 3.4627 | 3.6835 | 3.4050 | 3.2600 | 3.3198 | 2.7337 | 2.4913 | 3.1500 | 3.1400 | 3.0930 | 2.8867 |
| Maximum | 4.0520 | 3.9928 | 5.1978 | 4.2891 | 3.6965 | 4.4900 | 3.7952 | 3.6174 | 4.4669 | 5.2570 | 4.2101 | 4.3088 |
| **Calcium (mg/L)** | **FPM1** | **FPM2** | **FPM3** | **FPM4** | **FPM5** | **FPM6** | **FPM7** | **FPM8** | **FPM9** | **FPM10** | **FPM11** | **FPM12** |
| Minimum | 10.596 | 9.7351 | 10.968 | 8.1792 | 8.9930 | 8.8255 | 9.7111 | 9.3401 | 10.740 | 11.200 | 11.327 | 11.004 |
| 25th percentile | 19.935 | 20.687 | 24.280 | 19.131 | 16.495 | 13.600 | 18.391 | 14.600 | 16.775 | 13.275 | 17.725 | 16.050 |
| Median | 28.639 | 22.651 | 27.714 | 20.050 | 20.912 | 21.100 | 25.100 | 15.300 | 19.542 | 16.800 | 21.200 | 17.300 |
| Mean | 26.628 | 22.770 | 28.167 | 22.346 | 21.304 | 21.171 | 23.357 | 17.665 | 21.307 | 19.348 | 21.447 | 17.816 |
| 75th percentile | 31.900 | 26.038 | 34.550 | 23.375 | 23.525 | 26.100 | 29.250 | 21.528 | 20.900 | 25.125 | 24.100 | 18.864 |
| 90th percentile | 32.100 | 30.220 | 35.600 | 31.370 | 34.760 | 32.320 | 30.750 | 25.120 | 37.040 | 26.646 | 28.100 | 21.736 |
| Maximum | 43.800 | 32.200 | 41.200 | 41.400 | 37.300 | 43.300 | 35.900 | 28.800 | 39.100 | 34.400 | 34.000 | 28.900 |
| **Magnesium (mg/L)** | **FPM1** | **FPM2** | **FPM3** | **FPM4** | **FPM5** | **FPM6** | **FPM7** | **FPM8** | **FPM9** | **FPM10** | **FPM11** | **FPM12** |
| Minimum | 1.9554 | 1.5928 | 2.1506 | 0.0500 | 0.0500 | 1.1256 | 0.9722 | 0.0500 | 1.7741 | 1.8700 | 0.0500 | 1.0419 |
| 25th percentile | 2.6181 | 1.8724 | 2.8473 | 1.8750 | 2.2235 | 2.6965 | 2.7752 | 2.0320 | 2.3243 | 3.2901 | 2.4048 | 2.2779 |
| Median | 2.9850 | 2.7493 | 3.6900 | 2.8800 | 2.9600 | 3.2898 | 3.4948 | 2.5000 | 2.8800 | 3.6703 | 3.0861 | 3.4600 |
| Mean | 3.3570 | 2.8138 | 3.4236 | 2.7781 | 2.8277 | 3.2772 | 3.3852 | 2.5111 | 2.9590 | 3.9463 | 2.9734 | 3.2231 |
| 75th percentile | 4.0224 | 3.6025 | 3.8966 | 3.4950 | 3.5075 | 3.7655 | 3.7927 | 2.9100 | 3.3781 | 4.2531 | 3.4975 | 3.9500 |
| 90th percentile | 4.9414 | 4.0700 | 4.1624 | 4.4616 | 4.1195 | 4.8200 | 4.4403 | 3.6905 | 3.8249 | 5.6670 | 4.0106 | 4.4749 |
| Maximum | 5.3401 | 4.1826 | 5.0813 | 4.9200 | 4.7645 | 5.2377 | 6.0803 | 4.3554 | 5.4063 | 7.5609 | 6.1586 | 5.3762 |

The limits of quantification (LOQ) were as follows: TOC: 0.5 mg/l; DOC: 0.5 mg/L; Fluoride: 0.05 mg/L; Chloride: 1 mg/L; Nitrite: 0.001 mg/L; Nitrate: 0.1 mg/L; Phosphate: 0.05 mg/L; Sulfate: 1 mg/L; Sodium: 0.1 mg/L; Ammonium: 0.02 mg/L; Potassium: 0.1 mg/L; Calcium: 0.1 mg/L; Magnesium: 0.1 mg/L.

**Table S6.** Statistical characteristics of the physico-chemical parameters specific conductivity, pH, water temperature and dissolved oxygen measured for FPM 1-12 during the study period (06 November 2017 – 31 July 2019).

| **Specific conductivity (µS/cm)** | **FPM1** | **FPM2** | **FPM3** | **FPM4** | **FPM5** | **FPM6** | **FPM7** | **FPM8** | **FPM9** | **FPM10** | **FPM11** | **FPM12** |
| --- | --- | --- | --- | --- | --- | --- | --- | --- | --- | --- | --- | --- |
| Minimum | 18.3 | 21.6 | 98.3 | 88.1 | 87.5 | 87.9 | 98.6 | 82.7 | 90.4 | 86.6 | 86.6 | 90.0 |
| 25th percentile | 132.0 | 122.5 | 162.0 | 115.5 | 110.1 | 100.3 | 145.2 | 97.2 | 120.5 | 139.5 | 122.7 | 103.5 |
| Median | 179.9 | 143.5 | 166.6 | 151.1 | 131.9 | 130.0 | 169.8 | 108.4 | 120.3 | 117.5 | 146.9 | 115.0 |
| 75th percentile | 206.0 | 176.5 | 188.4 | 177.4 | 160.5 | 159.1 | 193.0 | 128.2 | 135.0 | 154.2 | 174.0 | 171.9 |
| 90th percentile | 274.1 | 223.9 | 222.0 | 252.9 | 185.6 | 201.4 | 207.3 | 170.5 | 158.0 | 206.5 | 189.6 | 205.5 |
| Maximum | 281.0 | 298.0 | 290.0 | 330.0 | 253.4 | 281.0 | 281.4 | 224.5 | 272.1 | 299.4 | 283.5 | 264.2 |
| Mean | 182.8 | 155.8 | 174.5 | 163.1 | 140.5 | 142.3 | 170.3 | 121.3 | 126.5 | 137.0 | 149.6 | 137.8 |
| **pH** | **FPM1** | **FPM2** | **FPM3** | **FPM4** | **FPM5** | **FPM6** | **FPM7** | **FPM8** | **FPM9** | **FPM10** | **FPM11** | **FPM12** |
| Minimum | 7.1 | 7.3 | 7.4 | 7.2 | 7.5 | 7.3 | 7.2 | 7.6 | 7.5 | 7.8 | 7.3 | 6.7 |
| 25th percentile | 8.1 | 8.3 | 8.1 | 8.0 | 8.1 | 8.1 | 8.2 | 8.2 | 8.0 | 8.1 | 8.4 | 8.0 |
| Median | 8.2 | 8.7 | 8.1 | 8.3 | 8.6 | 8.3 | 8.4 | 9.0 | 8.8 | 8.9 | 8.8 | 9.0 |
| 75th percentile | 8.4 | 9.1 | 8.3 | 8.8 | 8.8 | 8.7 | 8.6 | 9.3 | 9.3 | 9.5 | 9.2 | 9.6 |
| 90th percentile | 8.9 | 9.4 | 8.6 | 9.0 | 9.1 | 8.9 | 8.9 | 9.4 | 9.7 | 9.7 | 9.4 | 9.9 |
| Maximum | 9.7 | 9.7 | 9.1 | 10.0 | 9.9 | 9.1 | 9.3 | 10.2 | 9.8 | 10.0 | 10.1 | 10.3 |
| Mean | 8.1 | 8.6 | 8.1 | 8.4 | 8.5 | 8.3 | 8.3 | 8.8 | 8.8 | 8.9 | 8.7 | 8.8 |
| **Temperature (°C)** | **FPM1** | **FPM2** | **FPM3** | **FPM4** | **FPM5** | **FPM6** | **FPM7** | **FPM8** | **FPM9** | **FPM10** | **FPM11** | **FPM12** |
| Minimum | 2.6 | 2.6 | 2.5 | 2.3 | 2.4 | 2.4 | 2.7 | 2.6 | 6.7 | 2.9 | 3.4 | 3.3 |
| 25th percentile | 6.4 | 6.5 | 6.6 | 6.5 | 6.5 | 6.6 | 6.8 | 6.5 | 16.8 | 5.4 | 5.9 | 7.3 |
| Median | 11.7 | 12.4 | 13.6 | 12.8 | 13.1 | 13.4 | 12.9 | 12.8 | 19.7 | 11.3 | 15.5 | 15.3 |
| 75th percentile | 19.1 | 19.1 | 19.5 | 19.4 | 18.6 | 19.4 | 19.2 | 18.7 | 22.3 | 19.7 | 20.2 | 18.9 |
| 90th percentile | 22.6 | 22.6 | 22.9 | 23.0 | 21.9 | 22.8 | 22.6 | 22.2 | 23.6 | 22.7 | 22.5 | 21.9 |
| Maximum | 26.1 | 25.6 | 25.8 | 26.0 | 25.5 | 25.7 | 26.0 | 25.6 | 25.4 | 25.2 | 24.7 | 24.5 |
| Mean | 12.9 | 13.1 | 13.6 | 13.3 | 13.1 | 13.5 | 13.3 | 13.1 | 18.9^*^ | 12.6^*^ | 13.6^*^ | 13.7^*^ |
| **Oxygen (mg/L)** | **FPM1** | **FPM2** | **FPM3** | **FPM4** | **FPM5** | **FPM6** | **FPM7** | **FPM8** | **FPM9** | **FPM10** | **FPM11** | **FPM12** |
| Minimum | 0.1 | 0.1 | 2.0 | 0.1 | 1.4 | 1.5 | 0.6 | 2.2 | 1.2 | 1.2 | 0.1 | 0.1 |
| 25^th^ percentile | 6.5 | 8.2 | 8.3 | 7.4 | 8.7 | 8.9 | 8.3 | 9.0 | 6.9 | 8.6 | 7.8 | 6.7 |
| Median | 9.9 | 10.9 | 9.8 | 10.1 | 10.7 | 10.9 | 10.5 | 12.2 | 8.6 | 10.6 | 9.9 | 9.3 |
| 75^th^ percentile | 12.4 | 14.0 | 11.4 | 12.6 | 12.5 | 12.1 | 12.6 | 14.1 | 10.0 | 13.1 | 13.2 | 11.4 |
| 90^th^ percentile | 14.6 | 16.3 | 12.9 | 13.8 | 14.4 | 13.4 | 14.2 | 15.8 | 10.6 | 16.8 | 17.2 | 13.2 |
| Maximum | 17.5 | 26.8 | 19.5 | 15.9 | 18.5 | 16.5 | 19.0 | 22.1 | 12.1 | 22.5 | 24.8 | 20.5 |
| Mean | 9.3 | 11.1 | 9.9 | 9.4 | 10.6 | 10.5 | 10.2 | 11.8 | 8.2^*^ | 11.0^*^ | 10.2^*^ | 8.7^*^ |

Please note that water temperature and dissolved oxygen data are missing due to logger failure (defective devices) for the following FPMs and time spans: FPM 1-12: 22 December 2017 – 22 March 2018; FPM9: 06 November 2017 – 20 December 2017 & 10 August 2018 – 20 June 2019; FPM10: 10 August 2018 – 07 January 2019 & 22 March 2019 – 31 July 2019; FPM11: 10 August 2018 – 07 January 2019 & 22 March 2019 – 20 June 2019; FPM12: 15 September 2018 – 10 January 2019. The mean values for water temperature and dissolved oxygen of FPM 9-12 (indicated by an asterisk) should be interpreted with care due to high amounts of missing values.

**Colonization of aquatic and terrestrial ecosystem compartments**

**Merolimnic insect emergence**

Overall, 3345 emerged individuals have been captured in 2019 and identified at the family level; the individuals belong to nine families (*Chironomidae, Baetidae, Coenagrionidae, Culicidae, Chaoboridae, Libellulidae, Tipulidae, Sialidae, Limnephilidae*) from six orders. The numbers of emerged families increased across all ponds from end of April 2019 (mean of 0.7 families) till mid of June 2019 (mean number of 2.1 families), followed by a decrease till end of July 2019 (mean number of 0.9 families) (Figure S8). Concerning individual FPMs, the highest richness of emerged families (n = 5) per sampling date was recorded for FPM1 at the end of June 2019, while, in contrast, a maximum of one emerged family only could concurrently be found for eight of the FPMs.

Across all sampling dates, FPM7 had the overall highest family richness (n = 8), whereas only two different families were recorded for FPM8 and FPM12 (Table S7). The total number of individuals also differed substantially between the ponds, with highest numbers found for FPM3 (n = 843 individuals) and lowest numbers found for FPM12 (n = 134 individuals) (Table S7). Individuals belonging to the families *Chironomidae* (81.2%), *Baetidae* (16.04%) and *Coenagrionidae* (2.14%) were most often detected.

**Figure S8.** Development of the no. of families of emerged merolimnic insects sampled in FPM 1-12 in 2019. Black circles depict means and error bars show 95% confidence intervals.

**Table S7.** Detailed results of the macroinvertebrate, merolimnic insect emergence, zooplankton, amphibian and submerged vegetation assessments for FPM 1-12 during the study period (06 November 2017 – 31 July 2019).

| **No. of macroinvertebrate families** | **FPM1** | **FPM2** | **FPM3** | **FPM4** | **FPM5** | **FPM6** | **FPM7** | **FPM8** | **FPM9** | **FPM10** | **FPM11** | **FPM12** |
| --- | --- | --- | --- | --- | --- | --- | --- | --- | --- | --- | --- | --- |
| 11 December 17 | 2 | 2 | 0 | 0 | 0 | 1 | 0 | 1 | 1 | 0 | 1 | 0 |
| 05 February 18 | 2 | 2 | 1 | 1 | 1 | 0 | 2 | 1 | 1 | 2 | 1 | 3 |
| 02 May 18 | 3 | 2 | 4 | 3 | 3 | 3 | 3 | 2 | 3 | 2 | 2 | 2 |
| 29 May 18 | 1 | 3 | 4 | 3 | 2 | 3 | 3 | 2 | 4 | 1 | 3 | 2 |
| 27 June 18 | 7 | 2 | 2 | 5 | 5 | 4 | 4 | 2 | 2 | 4 | 2 | 6 |
| 01 August 18 | 7 | 5 | 6 | 4 | 7 | 7 | 4 | 4 | 5 | 7 | 1 | 11 |
| 22 August 18 | 9 | 6 | 6 | 5 | 4 | 7 | 6 | 6 | 6 | 8 | 8 | 10 |
| 24 September 18 | 7 | 4 | 10 | 4 | 3 | 7 | 8 | 4 | 8 | 6 | 3 | 7 |
| 18 October 18 | 4 | 3 | 6 | 4 | 3 | 7 | 4 | 4 | 1 | 3 | 3 | 4 |
| 16 November 18 | 6 | 3 | 8 | 2 | 4 | 8 | 3 | 6 | 4 | 7 | 4 | 7 |
| 14 December 18 | 5 | 2 | 8 | 5 | 3 | 10 | 5 | 5 | 8 | 5 | 3 | 5 |
| 11 January 19 | 5 | 6 | 6 | 4 | 2 | 4 | 8 | 4 | 4 | 11 | 2 | 3 |
| 15 March 19 | 4 | 4 | 6 | 5 | 4 | 7 | 6 | 5 | 3 | 5 | 4 | 7 |
| 02 May 19 | 5 | 2 | 8 | 4 | 4 | 10 | 4 | 5 | 6 | 7 | 3 | 4 |
| 27 June 19 | 6 | 5 | 7 | 3 | 4 | 8 | 6 | 5 | 3 | 4 | 4 | 7 |
| Total no. of different families (2017 - 2019) | 17 | 14 | 17 | 14 | 13 | 17 | 17 | 13 | 17 | 17 | 11 | 19 |
| Total no. of individuals (2017 - 2019) | 841 | 421 | 1052 | 417 | 332 | 693 | 451 | 446 | 614 | 806 | 431 | 1494 |
| **No of emerged families** | **FPM1** | **FPM2** | **FPM3** | **FPM4** | **FPM5** | **FPM6** | **FPM7** | **FPM8** | **FPM9** | **FPM10** | **FPM11** | **FPM12** |
| 24 April 19 | 0 | 0 | 1 | 0 | 1 | 2 | 1 | 0 | 1 | 1 | 0 | 1 |
| 30 April 19 | 1 | 1 | 1 | 1 | 1 | 1 | 2 | 1 | 1 | 2 | 1 | 1 |
| 08 May 19 | 1 | 1 | 1 | 1 | 1 | 1 | 2 | 1 | 2 | 1 | 1 | 1 |
| 15 May 19 | 1 | 1 | 1 | 1 | 1 | 2 | 1 | 1 | 1 | 1 | 1 | 1 |
| 22 May 19 | 3 | 1 | 3 | 1 | 1 | 1 | 3 | 1 | 3 | 2 | 2 | 1 |
| 29 May 19 | 4 | 3 | 4 | 1 | 1 | 1 | 1 | 1 | 1 | 2 | 4 | 1 |
| 04 June 19 | 2 | 1 | 4 | 2 | 2 | 2 | 2 | 1 | 1 | 1 | 1 | 1 |
| 12 June 19 | 4 | 4 | 1 | 1 | 1 | 2 | 4 | 1 | 0 | 1 | 4 | 2 |
| 19 June 19 | 3 | 1 | 1 | 0 | 1 | 1 | 3 | 0 | 2 | 1 | 1 | 0 |
| 29 June 19 | 5 | 2 | 1 | 4 | 1 | 1 | 2 | 1 | 1 | 0 | 1 | 1 |
| 03 July 19 | 2 | 0 | 3 | 2 | 1 | 1 | 3 | 1 | 2 | 1 | 2 | 1 |
| 09 July 19 | 2 | 1 | 2 | 1 | 1 | 1 | 3 | 2 | 1 | 0 | 3 | 1 |
| 17 July 19 | 1 | 1 | 2 | 2 | 1 | 1 | 0 | 1 | 1 | 1 | 1 | 1 |
| 22 July 19 | 0 | 1 | 2 | 0 | 2 | 1 | 1 | 0 | 1 | 1 | 1 | 1 |
| 31 July 19 | 0 | 0 | 1 | 2 | 1 | 1 | 2 | 0 | 1 | 1 | 1 | 1 |
| Total no. of different families in 2019 | 5 | 4 | 5 | 4 | 3 | 3 | 8 | 2 | 3 | 3 | 5 | 2 |
| Total no. of individuals in 2019 | 437 | 159 | 843 | 152 | 272 | 167 | 436 | 136 | 221 | 190 | 196 | 134 |
| **No. of zooplankton individuals per sample** | **FPM1** | **FPM2** | **FPM3** | **FPM4** | **FPM5** | **FPM6** | **FPM7** | **FPM8** | **FPM9** | **FPM10** | **FPM11** | **FPM12** |
| 15 November 17 | 0 |  |  | 13 | 0 |  | 11 | 17 | 1 | 1 |  | 2 |
| 18 December 17 |  |  |  | 0 | 5 | 3 | 1 | 0 | 0 | 20 | 0 | 1 |
| 12 March 18 |  | 1 |  | 4 | 3 | 0 | 4 | 18 | 0 | 48 | 1 | 1 |
| 05 April 18 | 10 | 8 |  | 2 | 2 | 3 | 17 | 12 | 71 |  | 75 | 3 |
| 28 May 18 |  |  | 0 | 0 | 0 | 0 | 0 | 0 |  |  |  | 1 |
| 26 June 18 | 13 | 482 | 1021 | 162 | 9 | 8 | 8 |  | 186 | 393 | 972 | 4048 |
| 11 July 18 | 5 | 525 | 3 | 0 | 19 | 8 | 0 | 8 | 76 | 6896 | 127 | 6800 |
| 21 August 18 | 656 | 840 | 72 | 2160 | 2224 | 163 | 1120 | 10560 | 1408 | 1600 | 792 | 628 |
| 17 September 18 | 576 | 2260 | 31 | 115 | 493 | 25 | 520 | 1336 | 264 | 572 | 5056 | 1808 |
| 02 October 18 | 26 | 1184 | 54 | 50 | 1752 | 92 | 868 | 1624 | 588 | 416 | 564 | 296 |
| 06 November 18 | 32 | 45 | 68 | 624 | 112 | 100 | 390 | 30 | 252 | 134 | 596 | 72 |
| 04 December 18 | 41 | 650 | 14 | 137 | 900 | 5 | 140 | 2996 | 564 | 68 | 672 | 65 |
| 17 January 19 |  | 38 | 5 | 142 | 304 | 28 | 100 | 224 |  | 58 | 52 | 48 |
| 11 February 19 | 4 | 34 | 36 | 20 | 25 | 46 | 92 | 128 | 352 | 256 | 816 | 384 |
| 12 March 19 | 456 | 59 | 28 | 30 | 376 | 81 | 27 | 21 | 124 | 384 | 688 | 1632 |
| 29 April 19 | 2272 | 968 | 42 | 864 | 21 | 176 | 1312 | 1376 | 992 | 1608 | 1120 | 1856 |
| 23 May 19 | 3040 | 3392 | 160 | 2176 | 2688 | 70 | 832 | 2816 | 784 | 2368 | 0 | 2112 |
| 11 June 19 | 640 | 2560 | 1280 | 496 | 384 | 1920 | 5376 | 1792 | 6016 | 3520 | 4224 | 3072 |
| 10 July 19 | 832 | 3456 | 640 | 2496 | 704 | 320 | 992 | 1184 | 1120 | 1344 | 2048 | 2816 |
| Overall mean | 574 | 1031 | 230 | 500 | 527 | 169 | 622 | 1341 | 753 | 1158 | 1047 | 1350 |
| **Tadpole coverage (%)** | **FPM1** | **FPM2** | **FPM3** | **FPM4** | **FPM5** | **FPM6** | **FPM7** | **FPM8** | **FPM9** | **FPM10** | **FPM11** | **FPM12** |
| 19 April 18 | 7.66 | 29.52 | 10.61 | 6.66 | 5.25 | 9.21 | 20.25 | 6.93 | 9.85 | 11.40 | 26.16 | 23.02 |
| 03 May 18 | 41.50 | 19.55 | 28.01 | 18.84 | 23.83 | 37.29 | 33.73 | 31.60 | 41.14 | 14.44 | 20.10 | 23.60 |
| 22 May 18 | 20.92 | 3.80 | 19.51 | 17.72 | 29.56 | 30.60 | 33.92 | 25.24 | 28.65 | 6.44 | 0.00 | 1.10 |
| 07 June 18 | 8.15 | 0.00 | 14.34 | 9.24 | 19.71 | 11.16 | 32.27 | 45.48 | 29.17 | 38.17 | 3.15 | 1.45 |
| 22 March 19 | 0.00 | 0.00 | 0.00 | 0.00 | 0.00 | 0.00 | 0.00 | 0.00 | 0.00 | 0.00 | 0.00 | 0.00 |
| 26 March 19 | 0.00 | 0.00 | 0.00 | 0.00 | 0.00 | 0.00 | 0.00 | 0.00 | 0.00 | 0.00 | 0.00 | 0.00 |
| 03 April 19 | 2.92 | 9.28 | 2.33 | 15.74 | 6.37 | 2.83 | 0.00 | 0.64 | 0.68 | 0.00 | 1.32 | 3.64 |
| 05 April 19 | 2.58 | 9.48 | 0.00 | 2.50 | 20.49 | 2.18 | 2.75 | 7.21 | 0.86 | 0.00 | 4.00 | 3.77 |
| 12 April 19 | 9.57 | 14.70 | 0.00 | 47.25 | 27.61 | 1.02 | 0.00 | 0.00 | 0.00 | 13.63 | 6.65 | 5.96 |
| 15 April 19 | 26.70 | 25.45 | 8.06 | 26.77 | 70.13 | 2.90 | 5.18 | 8.50 | 3.73 | 9.21 | 10.66 | 2.26 |
| 18 April 19 | 24.36 | 2.21 | 4.03 | 43.36 | 68.54 | 0.00 | 1.51 | 7.15 | 0.62 | 9.57 | 7.31 | 13.89 |
| 23 April 19 | 76.53 | 16.96 | 18.96 | 34.18 | 71.24 | 0.00 | 3.80 | 14.35 | 4.34 | 3.69 | 8.91 | 5.05 |
| 26 April 19 | 90.59 | 49.74 | 17.47 | 40.16 | 79.04 | 0.00 | 2.20 | 54.42 | 5.16 | 9.53 | 14.56 | 17.06 |
| 29 April 19 | 87.45 | 37.45 | 34.20 | 25.77 | 59.44 | 0.00 | 17.14 | 79.56 | 42.00 | 12.32 | 38.96 | 27.58 |
| 03 May 19 | 84.44 | 59.05 | 65.24 | 36.33 | 73.96 | 0.00 | 7.45 | 54.70 | 44.32 | 9.39 | 42.42 | 30.72 |
| 07 May 19 | 67.66 | 49.11 | 31.83 | 42.87 | 45.64 | 0.33 | 0.00 | 40.52 | 29.01 | 10.61 | 31.16 | 28.02 |
| 10 May 19 | 69.03 | 34.66 | 53.82 | 42.80 | 54.42 | 0.00 | 11.63 | 53.13 | 36.97 | 14.69 | 38.43 | 35.61 |
| 17 May 19 | 39.55 | 39.35 | 37.23 | 37.39 | 39.49 | 0.00 | 0.00 | 42.24 | 32.80 | 14.89 | 35.40 | 34.09 |
| 23 May 19 | 43.76 | 34.03 | 36.79 | 33.49 | 32.72 | 0.00 | 34.91 | 38.97 | 25.76 | 12.89 | 41.98 | 44.01 |
| 31 May 19 | 8.11 | 0.00 | 36.15 | 12.10 | 18.20 | 0.00 | 0.00 | 10.36 | 27.67 | 15.30 | 0.00 | 0.00 |
| 07 June 19 | 33.29 | 22.32 | 25.74 | 29.31 | 25.68 | 27.82 | 28.88 | 32.94 | 0.00 | 6.64 | 0.00 | 12.98 |
| 14 June 19 | 42.80 | 0.07 | 0.08 | 0.24 | 0.85 | 0.00 | 0.47 | 0.47 | 0.31 | 2.09 | 0.00 | 0.08 |
| Overall mean | 35.80 | 20.76 | 20.20 | 23.76 | 35.10 | 5.70 | 10.73 | 25.20 | 16.50 | 9.77 | 15.05 | 14.27 |
| **Submerged vegetation coverage (%)** | **FPM1** | **FPM2** | **FPM3** | **FPM4** | **FPM5** | **FPM6** | **FPM7** | **FPM8** | **FPM9** | **FPM10** | **FPM11** | **FPM12** |
| 31 January 18 | 2.10 | 7.85 | 5.49 | 1.09 | 8.61 | 5.84 | 8.58 | 4.56 | 4.37 | 12.59 | 10.63 | 6.02 |
| 20 February 18 | 0.00 | 10.69 | 1.36 | 1.26 | 9.35 | 5.25 | 11.93 | 9.73 | 3.37 | 7.83 | 17.08 | 10.14 |
| 08 March 18 | 0.00 | 4.33 | 0.74 | 1.46 | 7.21 | 2.72 | 6.79 | 4.70 | 3.29 | 27.83 | 13.90 | 4.67 |
| 22 March 18 | 3.30 | 0.00 | 0.95 | 1.73 | 5.32 | 0.00 | 3.15 | 2.23 | 0.10 | 6.87 | 17.54 | 0.31 |
| 05 April 18 | 0.44 | 0.78 | 1.04 | 0.25 | 1.50 | 0.00 | 3.25 | 1.15 | 0.00 | 1.63 | 2.37 | 0.00 |
| 03 May 18 | 0.00 | 0.00 | 0.00 | 8.42 | 0.00 | 0.00 | 0.00 | 0.00 | 0.00 | 11.37 | 0.00 | 0.00 |
| 22 May 18 | 0.00 | 1.56 | 0.00 | 0.67 | 15.26 | 0.00 | 0.00 | 0.00 | 4.78 | 1.69 | 0.00 | 11.43 |
| 08 June 18 | 4.35 | 7.31 | 1.60 | 4.26 | 10.65 | 2.90 | 5.61 | 2.84 | 3.47 | 1.29 | 0.10 | 0.00 |
| 13 July 18 | 19.33 | 30.65 | 3.55 | 27.74 | 20.87 | 22.83 | 12.28 | 17.34 | 21.30 | 6.55 | 14.23 | 13.02 |
| 20 August 18 | 44.67 | 36.09 | 9.17 | 57.86 | 44.78 | 19.36 | 30.83 | 53.80 | 35.02 | 5.33 | 6.46 | 45.08 |
| 27 August 18 | 44.82 | 36.80 | 8.13 | 60.12 | 49.91 | 16.85 | 28.41 | 57.34 | 34.65 | 22.24 | 59.43 | 50.37 |
| 31 August 18 | 48.40 | 50.95 | 11.29 | 75.69 | 56.38 | 23.73 | 31.65 | 69.76 | 43.12 | 26.11 | 63.05 | 59.95 |
| 10 September 18 | 48.60 | 56.31 | 15.06 | 79.67 | 71.93 | 26.49 | 36.92 | 66.60 | 41.12 | 22.83 | 68.67 | 59.33 |
| 28 September 18 | 58.26 | 67.75 | 14.09 | 75.03 | 58.50 | 30.32 | 35.80 | 72.36 | 52.96 | 34.78 | 68.48 | 67.20 |
| 29 October 18 | 58.58 | 70.70 | 15.24 | 72.89 | 55.88 | 26.37 | 40.47 | 71.65 | 38.45 | 36.43 | 73.04 | 65.68 |
| 16 November 18 | 55.22 | 76.52 | 12.30 | 67.62 | 60.95 | 24.47 | 36.82 | 69.18 | 33.23 | 32.02 | 74.59 | 48.50 |
| 30 November 18 | 52.11 | 70.00 | 11.35 | 73.74 | 65.21 | 22.80 | 34.46 | 63.16 | 41.21 | 35.25 | 68.50 | 70.76 |
| 21 December 18 | 57.93 | 76.97 | 13.08 | 73.97 | 67.49 | 29.52 | 33.74 | 64.36 | 40.01 | 40.19 | 71.60 | 73.97 |
| 04 January 19 | 61.61 | 84.65 | 11.21 | 74.92 | 71.27 | 32.21 | 41.14 | 70.16 | 43.30 | 42.22 | 72.89 | 79.23 |
| 08 February 19 | 46.69 | 59.01 | 7.57 | 64.89 | 60.67 | 23.12 | 34.80 | 53.82 | 35.87 | 37.07 | 64.62 | 67.53 |
| 20 March 19 | 63.26 | 91.81 | 23.33 | 82.50 | 80.31 | 36.23 | 40.28 | 82.20 | 43.67 | 43.56 | 86.74 | 83.13 |
| 03 April 19 | 51.30 | 82.39 | 21.16 | 69.39 | 67.55 | 34.15 | 45.64 | 70.77 | 48.06 | 45.71 | 79.40 | 70.16 |
| 17 April 19 | 58.08 | 87.93 | 19.16 | 69.35 | 68.32 | 35.66 | 50.11 | 68.47 | 57.49 | 46.11 | 74.76 | 69.24 |
| 30 April 19 | 60.87 | 66.49 | 39.83 | 73.50 | 66.19 | 40.20 | 49.12 | 66.55 | 62.64 | 44.03 | 66.85 | 67.18 |
| 15 May 19 | 68.72 | 78.56 | 23.82 | 67.23 | 74.16 | 51.47 | 54.82 | 71.64 | 54.63 | 49.18 | 73.78 | 76.34 |
| 29 May 19 | 84.78 | 84.20 | 24.59 | 70.66 | 75.96 | 53.59 | 56.76 | 67.48 | 67.34 | 52.69 | 70.00 | 73.65 |
| 12 June 19 | 81.90 | 84.72 | 36.09 | 80.69 | 70.93 | 39.22 | 57.16 | 73.67 | 76.35 | 59.07 | 75.09 | 74.15 |
| 25 June 19 | 86.81 | 82.54 | 44.03 | 76.30 | 77.51 | 54.37 | 69.67 | 75.53 | 75.17 | 69.16 | 71.43 | 83.43 |
| 10 July 19 | 84.14 | 87.63 | 45.30 | 86.81 | 84.14 | 93.67 | 80.87 | 84.30 | 85.31 | 80.30 | 85.62 | 85.55 |
| 23 July 19 | 89.65 | 81.04 | 47.39 | 87.65 | 90.49 | 84.67 | 88.61 | 92.92 | 81.10 | 83.83 | 86.70 | 83.93 |
| Overall mean | 44.53 | 52.54 | 15.60 | 52.91 | 49.91 | 27.93 | 34.32 | 50.28 | 37.71 | 32.86 | 51.25 | 50.00 |

**Table S8.** Results of the terrestrial vegetation survey at the FPMs in November 2018 (species present: 1; species absent: 0).

| **Species** | **FPM1** | **FPM2** | **FPM3** | **FPM4** | **FPM5** | **FPM6** | **FPM7** | **FPM8** | **FPM9** | **FPM10** | **FPM11** | **FPM12** |
| --- | --- | --- | --- | --- | --- | --- | --- | --- | --- | --- | --- | --- |
| *Agrostis stolonifera* | 1 | 1 | 1 | 1 | 1 | 1 | 1 | 1 | 1 | 1 | 1 | 1 |
| *Alisma plantago-aquatica* | 0 | 0 | 0 | 0 | 0 | 0 | 1 | 1 | 0 | 1 | 0 | 1 |
| *Alopecurus aequalis* | 1 | 1 | 1 | 1 | 1 | 0 | 1 | 0 | 0 | 1 | 1 | 0 |
| *Betula pendula* | 1 | 0 | 0 | 0 | 0 | 0 | 0 | 0 | 0 | 0 | 0 | 0 |
| *Carex acutiformes* | 1 | 1 | 0 | 1 | 0 | 0 | 0 | 0 | 0 | 0 | 0 | 0 |
| *Carex* sp. | 0 | 1 | 0 | 0 | 0 | 0 | 0 | 0 | 1 | 0 | 1 | 1 |
| *Carex* sp. | 0 | 1 | 0 | 0 | 1 | 0 | 0 | 0 | 0 | 0 | 0 | 0 |
| *Cerastium fontanum* | 0 | 0 | 0 | 0 | 0 | 0 | 1 | 0 | 0 | 0 | 0 | 1 |
| *Chenopodium album* | 0 | 1 | 0 | 0 | 0 | 0 | 0 | 0 | 0 | 0 | 0 | 0 |
| *Cichorium intybus* | 0 | 1 | 0 | 0 | 0 | 0 | 0 | 0 | 0 | 0 | 0 | 0 |
| *Cirsium palustre* | 0 | 1 | 0 | 0 | 0 | 0 | 0 | 0 | 0 | 1 | 0 | 1 |
| *Cyperus fuscus* | 0 | 1 | 0 | 0 | 0 | 0 | 0 | 0 | 0 | 0 | 0 | 0 |
| *Digitaria sanguinalis* | 0 | 0 | 0 | 0 | 0 | 0 | 0 | 1 | 0 | 0 | 0 | 0 |
| *Echinocloa crus-galli* | 0 | 1 | 0 | 1 | 0 | 0 | 0 | 1 | 1 | 1 | 1 | 1 |
| *Epilobium palustre* | 1 | 1 | 1 | 1 | 1 | 1 | 1 | 1 | 1 | 1 | 1 | 1 |
| *Equisetum arvense* | 1 | 1 | 0 | 0 | 1 | 0 | 1 | 0 | 1 | 0 | 0 | 0 |
| *Eragrostis minor* | 0 | 0 | 0 | 0 | 0 | 0 | 0 | 0 | 0 | 0 | 0 | 1 |
| *Erigeron annus* | 0 | 0 | 0 | 1 | 0 | 0 | 1 | 1 | 0 | 0 | 0 | 1 |
| *Eupatorium cannabinum* | 0 | 0 | 0 | 0 | 0 | 0 | 0 | 0 | 0 | 0 | 0 | 1 |
| *Euphorbia cyparissias* | 0 | 0 | 0 | 0 | 1 | 0 | 0 | 0 | 0 | 0 | 0 | 0 |
| *Glyceria fluitans* | 1 | 1 | 1 | 0 | 0 | 0 | 0 | 1 | 0 | 1 | 0 | 0 |
| *Gnaphalium uliginosum* | 0 | 0 | 0 | 1 | 0 | 0 | 1 | 0 | 1 | 0 | 1 | 0 |
| *Holcus lanatus* | 0 | 0 | 0 | 0 | 0 | 0 | 0 | 0 | 0 | 1 | 0 | 0 |
| *Isolepis setacea* | 1 | 0 | 1 | 0 | 0 | 1 | 0 | 0 | 1 | 1 | 0 | 1 |
| *Juncus effusus* | 1 | 1 | 1 | 1 | 1 | 1 | 1 | 1 | 1 | 1 | 1 | 1 |
| *Juncus tenuis* | 1 | 1 | 1 | 1 | 1 | 1 | 1 | 1 | 1 | 1 | 1 | 1 |
| *Juncus articulatus* | 1 | 1 | 1 | 1 | 1 | 1 | 1 | 1 | 1 | 1 | 1 | 1 |
| *Juncus bufonius* | 1 | 1 | 1 | 1 | 1 | 1 | 1 | 1 | 1 | 1 | 1 | 1 |
| *Lactuca serriola* | 0 | 0 | 0 | 0 | 0 | 0 | 0 | 1 | 0 | 0 | 0 | 1 |
| *Lolium perenne* | 1 | 1 | 1 | 1 | 0 | 0 | 0 | 1 | 0 | 1 | 0 | 0 |
| *Lycopus europaeus* | 0 | 1 | 0 | 1 | 0 | 0 | 0 | 1 | 0 | 0 | 0 | 0 |
| *Lythrum salicaria* | 0 | 1 | 0 | 0 | 0 | 1 | 1 | 0 | 0 | 0 | 0 | 1 |
| *Malva sylvestris* | 0 | 1 | 0 | 0 | 0 | 0 | 0 | 0 | 0 | 0 | 0 | 0 |
| *Medicago lupulina* | 1 | 1 | 0 | 0 | 1 | 1 | 1 | 1 | 1 | 1 | 1 | 1 |
| *Persicaria maculosa* | 0 | 1 | 0 | 1 | 0 | 0 | 0 | 1 | 1 | 0 | 1 | 1 |
| *Plantago lanceolata* | 0 | 0 | 0 | 1 | 1 | 1 | 1 | 0 | 0 | 1 | 0 | 0 |
| *Plantago major* | 0 | 1 | 1 | 1 | 1 | 0 | 1 | 1 | 0 | 0 | 1 | 1 |
| *Poa annua* | 0 | 0 | 0 | 0 | 0 | 0 | 0 | 0 | 0 | 0 | 0 | 1 |
| *Poa compressa* | 0 | 0 | 0 | 0 | 0 | 0 | 0 | 0 | 0 | 0 | 0 | 1 |
| *Prunella vulgaris* | 0 | 0 | 0 | 0 | 0 | 0 | 1 | 0 | 0 | 1 | 0 | 0 |
| *Ranunculus repens* | 1 | 1 | 1 | 0 | 1 | 0 | 0 | 1 | 0 | 0 | 1 | 0 |
| *Salix* sp. | 1 | 1 | 1 | 1 | 1 | 1 | 1 | 1 | 1 | 1 | 1 | 1 |
| *Salix caprea* | 0 | 1 | 1 | 1 | 0 | 1 | 1 | 0 | 0 | 0 | 0 | 1 |
| *Salix* sp. | 0 | 0 | 1 | 0 | 0 | 0 | 0 | 0 | 0 | 0 | 0 | 0 |
| *Solidago canadensis* | 0 | 1 | 0 | 0 | 0 | 0 | 0 | 0 | 0 | 0 | 0 | 0 |
| *Sonchus oleratius* | 1 | 1 | 0 | 0 | 0 | 0 | 1 | 0 | 0 | 0 | 0 | 0 |
| *Trifolium pratense* | 0 | 1 | 0 | 1 | 0 | 1 | 0 | 0 | 0 | 0 | 0 | 1 |
| *Trifolium repens* | 1 | 1 | 0 | 0 | 0 | 1 | 0 | 0 | 0 | 0 | 1 | 1 |
| *Tussilago farfara* | 0 | 0 | 1 | 0 | 0 | 0 | 0 | 1 | 0 | 0 | 1 | 1 |
| *Typha latifolia* | 1 | 1 | 1 | 1 | 1 | 1 | 1 | 1 | 1 | 1 | 1 | 1 |
| *Urtica dioica* | 0 | 0 | 0 | 0 | 1 | 0 | 0 | 0 | 0 | 0 | 0 | 0 |
| *Veronica beccabunga* | 1 | 1 | 1 | 0 | 1 | 1 | 0 | 0 | 0 | 0 | 0 | 0 |
| *Vicia hirsuta* | 1 | 0 | 0 | 0 | 1 | 1 | 0 | 0 | 0 | 0 | 0 | 0 |
| Total no. of species | 21 | 33 | 18 | 20 | 19 | 17 | 21 | 21 | 15 | 19 | 18 | 28 |

**Table S9.** Overall results of the ground beetle monitoring at the twelve FPMs for the sampling campaigns in May 2018, September/October 2018 and May 2019.

| **Species** | **FPM1** | **FPM2** | **FPM3** | **FPM4** | **FPM5** | **FPM6** | **FPM7** | **FPM8** | **FPM9** | **FPM10** | **FPM11** | **FPM12** |
| --- | --- | --- | --- | --- | --- | --- | --- | --- | --- | --- | --- | --- |
| *Acupalpis flavicollis* | 0 | 0 | 0 | 0 | 0 | 1 | 1 | 1 | 0 | 0 | 0 | 1 |
| *Acupalpus meridianus* | 0 | 0 | 0 | 0 | 1 | 0 | 0 | 0 | 0 | 0 | 0 | 0 |
| *Agonum emarginatum* | 5 | 0 | 1 | 11 | 1 | 1 | 0 | 6 | 7 | 3 | 2 | 3 |
| *Agonum fuliginosum* | 0 | 0 | 0 | 0 | 0 | 0 | 0 | 0 | 0 | 0 | 0 | 0 |
| *Agonum muelleri* | 2 | 0 | 0 | 0 | 0 | 0 | 0 | 0 | 1 | 0 | 0 | 1 |
| *Agonum sexpunctatum* | 41 | 10 | 20 | 16 | 16 | 21 | 7 | 26 | 12 | 11 | 14 | 18 |
| *Agonum viridicupreum* | 0 | 0 | 0 | 1 | 0 | 0 | 0 | 0 | 0 | 0 | 0 | 0 |
| *Amara aenea* | 1 | 0 | 1 | 0 | 0 | 0 | 1 | 0 | 0 | 0 | 0 | 0 |
| *Amara montivaga* | 0 | 0 | 0 | 0 | 0 | 0 | 0 | 0 | 0 | 0 | 0 | 0 |
| *Anisodactylus binotatus* | 1 | 0 | 0 | 0 | 0 | 0 | 0 | 0 | 0 | 1 | 0 | 0 |
| *Bembidion articulatum* | 1 | 0 | 0 | 2 | 0 | 1 | 4 | 0 | 1 | 0 | 1 | 1 |
| *Bembidion biguttatum* | 0 | 0 | 0 | 0 | 0 | 0 | 0 | 0 | 0 | 0 | 0 | 0 |
| *Bembidion bruxellense* | 0 | 0 | 0 | 0 | 0 | 0 | 1 | 0 | 0 | 0 | 0 | 0 |
| *Bembidion femoratum* | 0 | 1 | 1 | 0 | 1 | 1 | 0 | 0 | 0 | 0 | 1 | 0 |
| *Bembidion guttula* | 0 | 0 | 0 | 0 | 0 | 0 | 0 | 0 | 0 | 0 | 0 | 0 |
| *Bembidion illigeri* | 0 | 1 | 3 | 2 | 9 | 3 | 7 | 9 | 7 | 2 | 9 | 4 |
| *Bembidion lampros* | 0 | 0 | 0 | 0 | 0 | 0 | 0 | 0 | 0 | 0 | 0 | 0 |
| *Bembidion semipunctatum* | 0 | 0 | 1 | 0 | 0 | 0 | 0 | 0 | 0 | 0 | 0 | 0 |
| *Bembidion tetracolum* | 3 | 3 | 3 | 0 | 1 | 0 | 2 | 0 | 1 | 3 | 0 | 5 |
| *Carabus granulatus* | 0 | 1 | 0 | 0 | 0 | 0 | 0 | 0 | 0 | 0 | 0 | 0 |
| *Chlaenius nigricornis* | 0 | 0 | 0 | 0 | 0 | 0 | 0 | 0 | 1 | 0 | 1 | 1 |
| *Chlaenius vestitus* | 30 | 23 | 49 | 34 | 32 | 31 | 47 | 62 | 48 | 60 | 46 | 31 |
| *Cicindela campestris* | 2 | 0 | 1 | 0 | 1 | 0 | 2 | 1 | 0 | 0 | 0 | 2 |
| *Clivina collaris* | 0 | 0 | 0 | 1 | 0 | 0 | 0 | 0 | 0 | 0 | 0 | 0 |
| *Diachromus germanus* | 0 | 0 | 0 | 0 | 0 | 0 | 0 | 0 | 0 | 0 | 0 | 0 |
| *Dyschirius agnatus* | 0 | 0 | 1 | 1 | 0 | 0 | 1 | 0 | 0 | 0 | 1 | 1 |
| *Elaphrus cupreus* | 0 | 0 | 0 | 0 | 0 | 0 | 0 | 2 | 2 | 1 | 0 | 3 |
| *Elaphrus riparius* | 2 | 8 | 5 | 2 | 13 | 10 | 7 | 3 | 6 | 6 | 15 | 7 |
| *Harpalus rufipes* | 0 | 0 | 0 | 0 | 0 | 0 | 0 | 1 | 0 | 0 | 0 | 0 |
| *Loricera pilicornis* | 0 | 0 | 0 | 0 | 0 | 0 | 2 | 0 | 0 | 0 | 0 | 0 |
| *Nebria brevicollis* | 9 | 6 | 10 | 3 | 3 | 5 | 11 | 6 | 1 | 2 | 3 | 8 |
| *Nebria brevicollis* | 0 | 0 | 0 | 0 | 0 | 0 | 0 | 0 | 0 | 0 | 0 | 0 |
| *Oodes helopioides* | 0 | 0 | 0 | 1 | 0 | 0 | 0 | 2 | 0 | 0 | 0 | 1 |
| *Panagaeus cruxmajor* | 0 | 0 | 0 | 0 | 0 | 0 | 0 | 0 | 0 | 0 | 1 | 0 |
| *Paranchus albipes* | 1 | 5 | 3 | 4 | 5 | 2 | 4 | 3 | 28 | 20 | 16 | 17 |
| *Poecilus cupreus* | 0 | 1 | 0 | 0 | 0 | 0 | 0 | 0 | 0 | 0 | 0 | 0 |
| *Pterostichus diligens* | 0 | 0 | 0 | 0 | 0 | 0 | 0 | 0 | 0 | 0 | 0 | 0 |
| *Pterostichus minor* | 0 | 0 | 0 | 0 | 0 | 0 | 0 | 0 | 0 | 0 | 0 | 0 |
| *Pterostichus nigrita* | 0 | 0 | 0 | 0 | 0 | 0 | 0 | 1 | 1 | 1 | 2 | 4 |
| *Stenolophus mixtus* | 0 | 0 | 1 | 0 | 0 | 0 | 0 | 0 | 0 | 0 | 0 | 0 |
| *Stenolophus teutonus* | 35 | 11 | 2 | 11 | 4 | 23 | 24 | 2 | 10 | 13 | 19 | 8 |
| *Trechus obtusus* | 0 | 1 | 1 | 0 | 0 | 0 | 0 | 0 | 0 | 0 | 0 | 0 |
| Total no.of individuals | 133 | 71 | 103 | 89 | 87 | 99 | 121 | 125 | 126 | 123 | 131 | 116 |
| Total no. of species | 13 | 12 | 16 | 13 | 12 | 11 | 15 | 14 | 14 | 12 | 14 | 18 |

**Table S10.** Overview of the leafhopper species captured at FPM3, FPM5, FPM8 and FPM10 in June and September 2018.

| **Species** | **FPM3** | **FPM5** | **FPM8** | **FPM10** |
| --- | --- | --- | --- | --- |
| *Macrosteles sexnotatus* | 84 | 62 | 44 | 31 |
| *Arthaldeus pascuellus* | 27 | 39 | 18 | 38 |
| *Javesella pellucida* | 59 | 15 | 25 | 35 |
| *Deltocephalus pulicaris* | 69 | 12 | 13 | 32 |
| *Anaceratagallia ribauti* | 30 | 17 | 12 | 24 |
| *Cicadella viridis* | 2 | 2 | 6 | 12 |
| *Muelleriana fairmairei* | 0 | 1 | 1 | 0 |
| *Psammotettix helvolus* | 18 | 20 | 22 | 2 |
| *Psammotettix confinis* | 9 | 21 | 13 | 12 |
| *Zyginidia scutellaris* | 13 | 17 | 3 | 9 |
| *Megophthalmus scanicus* | 0 | 0 | 1 | 0 |
| *Laodelphax striatella* | 13 | 1 | 4 | 6 |
| *Cicadula sp. female* | 0 | 0 | 0 | 6 |
| *Javesella dubia* | 0 | 0 | 0 | 3 |
| *Allygus mixtus female* | 1 | 0 | 0 | 1 |
| *Emelyanoviana mollicula* | 0 | 2 | 0 | 0 |
| *Jassargus sp. female* | 0 | 0 | 0 | 1 |
| *Eupelix cuspidata* | 1 | 0 | 0 | 0 |
| *Megamelodes quadrimaculatus* | 0 | 0 | 1 | 0 |
| Total no. of species | 12 | 12 | 13 | 14 |
| Total no. of individuals | 326 | 209 | 163 | 212 |

**Aquatic and terrestrial litter decomposition**

**Table S11.** Overview of microbial (*k*_microbial_) and shredder-mediated (*k*_shredder_) leaf litter decomposition rates determined in the twelve FPMs. Please note that the sampling date indicates the end of the three-week sampling period.

| ***K*_microbial_** | **FPM1** | **FPM2** | **FPM3** | **FPM4** | **FPM5** | **FPM6** | **FPM7** | **FPM8** | **FPM9** | **FPM10** | **FPM11** | **FPM12** |
| --- | --- | --- | --- | --- | --- | --- | --- | --- | --- | --- | --- | --- |
| 30 May 18 | 0.0257 | 0.0284 | 0.0187 | 0.0177 | 0.0223 | 0.0242 | 0.0204 | 0.0190 | 0.0236 | 0.0246 | 0.0281 | 0.0282 |
| 28 June 18 | 0.0497 | 0.0473 | 0.0357 | 0.0496 | 0.0383 | 0.0286 | 0.0344 | 0.0286 | 0.0462 | 0.0373 | 0.0309 | 0.0293 |
| 31 July 18 | 0.0679 | 0.0585 | 0.0530 | 0.0657 | 0.0521 | 0.0627 | 0.0603 | 0.0511 | 0.0643 | 0.0397 | 0.0492 | 0.0647 |
| 24 August 18 | 0.0813 | 0.0383 | 0.0469 | 0.0447 | 0.0536 | 0.0508 | 0.0365 | 0.0444 | 0.0441 | 0.0390 | 0.0488 | 0.0368 |
| 21 September 18 | 0.0323 | 0.0350 | 0.0221 | 0.0307 | 0.0361 | 0.0391 | 0.0365 | 0.0357 | 0.0329 | 0.0300 | 0.0294 | 0.0303 |
| 19 November 18 | 0.0124 | 0.0117 | 0.0122 | 0.0089 | 0.0114 | 0.0093 |  | 0.0086 | 0.0051 | 0.0138 | 0.0159 | 0.0087 |
| 10 January 19 | 0.0119 | 0.0101 | 0.0153 | 0.0096 | 0.0123 | 0.0158 | 0.0160 | 0.0158 | 0.0152 | 0.0065 | 0.0081 | 0.0144 |
| 03 May 19 | 0.0173 | 0.0159 | 0.0165 | 0.0229 | 0.0200 | 0.0210 | 0.0184 | 0.0196 | 0.0179 | 0.0211 | 0.0180 | 0.0193 |
| 28 June 19 | 0.0178 | 0.0138 | 0.0235 | 0.0203 | 0.0201 | 0.0242 | 0.0239 | 0.0219 | 0.0193 | 0.0216 | 0.0246 | 0.0240 |
| Overall mean | 0.0351 | 0.0288 | 0.0271 | 0.0300 | 0.0296 | 0.0306 | 0.0308 | 0.0272 | 0.0298 | 0.0260 | 0.0281 | 0.0284 |
| ***k*_shredder_** | **FPM1** | **FPM2** | **FPM3** | **FPM4** | **FPM5** | **FPM6** | **FPM7** | **FPM8** | **FPM9** | **FPM10** | **FPM11** | **FPM12** |
| 30 May 18 | 0.0140 | 0.0156 | 0.0200 | 0.0206 | 0.0139 | 0.0096 | 0.0141 | 0.0129 | 0.0121 | 0.0100 | 0.0086 | 0.0097 |
| 28 June 18 | 0.0121 | 0.0126 | 0.0156 | 0.0114 | 0.0143 | 0.0211 | 0.0158 |  | 0.0121 | 0.0156 |  | 0.0175 |
| 31 July 18 | 0.0106 | 0.0114 | 0.0136 | 0.0096 | 0.0127 | 0.0100 | 0.0107 | 0.0123 | 0.0105 | 0.0175 | 0.0116 | 0.0112 |
| 24 August 18 | 0.0043 | 0.0118 | 0.0105 | 0.0101 | 0.0084 | 0.0103 | 0.0126 | 0.0093 | 0.0116 | 0.0119 | 0.0087 | 0.0140 |
| 21 September 18 | 0.0135 | 0.0120 | 0.0149 | 0.0124 | 0.0084 | 0.0098 | 0.0110 | 0.0113 | 0.0108 | 0.0136 | 0.0129 | 0.0118 |
| 19 November 18 | 0.0045 | 0.0094 | 0.0105 | 0.0120 | 0.0114 | 0.0040 |  | 0.0103 | 0.0152 | 0.0100 | 0.0091 | 0.0123 |
| 10 January 19 | 0.0109 | 0.0116 | 0.0119 | 0.0121 | 0.0112 | 0.0113 | 0.0108 | 0.0106 | 0.0111 | 0.0131 | 0.0123 | 0.0112 |
| 03 May 19 | 0.0104 | 0.0119 | 0.0115 | 0.0118 | 0.0111 | 0.0111 | 0.0110 | 0.0100 | 0.0105 | 0.0102 | 0.0095 | 0.0134 |
| 28 June 19 | 0.0136 | 0.0159 | 0.0140 | 0.0143 | 0.0102 | 0.0114 | 0.0129 | 0.0114 | 0.0118 | 0.0135 | 0.0106 | 0.0110 |
| Overall mean | 0.0104 | 0.0125 | 0.0136 | 0.0127 | 0.0113 | 0.0109 | 0.0124 | 0.0110 | 0.0118 | 0.0128 | 0.0104 | 0.0125 |

**Table S12**. Overview of the terrestrial litter decomposition expressed as linear weight loss per day (mg/day) for green tea and rooibos tea at the FPM banks. Please note that the sampling date indicates the end of the three-week sampling period.

| **Green tea** | **FPM1** | **FPM2** | **FPM3** | **FPM4** | **FPM5** | **FPM6** | **FPM7** | **FPM8** | **FPM9** | **FPM10** | **FPM11** | **FPM12** |
| --- | --- | --- | --- | --- | --- | --- | --- | --- | --- | --- | --- | --- |
| 12 December 17 | 33.86 | 23.80 | 27.99 | 29.84 | 35.61 | 28.96 | 28.48 | 30.54 | 28.78 | 30.37 | 33.17 | 27.73 |
| 21 February 18 | 20.37 | 33.63 | 29.71 | 27.78 | 29.51 | 27.45 | 25.57 | 27.85 | 22.16 | 24.02 | 20.85 | 29.23 |
| 04 April 18 | 29.30 | 29.62 | 31.48 | 32.75 | 33.68 | 29.07 | 29.95 | 32.53 | 32.25 | 28.17 | 32.64 | 27.73 |
| 28 June 18 | 44.01 | 45.08 | 43.30 | 41.90 | 42.67 | 43.83 | 45.16 | 45.09 | 44.31 | 47.82 | 43.73 | 41.57 |
| 02 August 18 | 19.91 | 27.27 | 28.31 | 17.97 | 27.94 | 27.76 | 25.57 | 27.36 | 30.41 | 34.03 | 32.78 | 32.12 |
| 27 August 18 | 40.68 | 42.79 | 43.47 | 48.62 | 51.70 | 45.56 | 43.62 | 47.79 | 41.79 | 43.36 | 43.74 | 42.81 |
| 19 September 18 | 48.97 |  | 50.88 | 47.35 | 51.67 | 49.76 | 43.60 | 50.68 | 47.39 | 48.56 | 41.22 | 50.96 |
| 19 December 18 | 27.65 | 31.34 | 29.50 | 29.17 | 28.86 | 28.85 | 28.09 | 31.71 | 30.61 | 27.82 | 28.97 | 34.06 |
| 14 January 19 | 28.33 | 28.99 | 27.34 | 29.44 | 30.14 | 29.63 | 27.99 | 38.09 | 31.72 | 31.75 | 27.76 | 29.01 |
| 12 March 19 | 25.64 | 24.96 | 23.84 | 24.50 | 27.85 | 25.21 | 24.70 | 26.84 | 27.50 | 24.60 | 25.55 | 25.62 |
| 03 May 19 | 34.54 | 36.30 | 37.52 | 35.25 | 37.21 | 36.49 | 36.08 | 37.31 | 37.39 | 34.32 | 32.98 | 34.35 |
| 22 June 19 | 41.24 | 41.90 | 40.78 | 43.44 | 42.53 | 34.98 | 41.61 | 41.58 | 41.47 | 37.50 | 39.35 | 39.69 |
| Overall mean | 32.87 | 33.24 | 34.51 | 34.00 | 36.61 | 33.96 | 33.37 | 36.45 | 34.65 | 34.36 | 33.56 | 34.57 |
| **Rooibos tea** | **FPM1** | **FPM2** | **FPM3** | **FPM4** | **FPM5** | **FPM6** | **FPM7** | **FPM8** | **FPM9** | **FPM10** | **FPM11** | **FPM12** |
| 12 December 17 | 17.11 | 17.60 | 16.21 | 20.34 | 17.58 | 16.82 | 20.18 | 18.18 | 16.82 | 16.38 | 15.02 | 16.57 |
| 21 February 18 | 15.60 | 20.55 | 18.58 | 18.37 | 17.67 | 17.99 | 19.84 |  | 14.77 | 17.57 | 14.45 | 14.02 |
| 04 April 18 | 20.42 | 20.26 | 20.11 | 20.44 | 19.96 | 21.20 | 19.06 | 18.30 | 21.88 | 19.18 | 20.43 | 21.50 |
| 28 June 18 | 21.37 | 25.66 | 26.05 | 20.12 | 19.77 | 24.89 | 20.01 | 29.20 | 25.99 | 25.67 | 27.59 | 26.60 |
| 02 August 18 | 9.84 | 13.41 | 9.90 | 9.95 | 12.22 | 19.58 | 8.92 | 14.44 | 15.06 | 18.88 | 18.62 | 16.39 |
| 27 August 18 | 16.70 | 20.33 | 23.47 | 18.09 | 19.15 | 19.47 | 19.29 | 20.57 | 21.41 | 27.66 | 28.31 | 28.04 |
| 19 September 18 | 16.85 | 19.55 | 12.30 | 15.55 | 16.68 | 23.37 | 22.49 | 19.71 | 22.49 | 23.07 | 18.62 | 20.18 |
| 19 December 18 | 9.01 | 9.84 | 10.71 | 11.11 | 12.33 | 15.27 | 11.15 | 16.04 | 12.54 | 12.27 | 10.67 | 9.89 |
| 14 January 19 | 12.95 | 12.06 | 10.98 | 11.40 | 12.22 | 10.99 | 11.98 | 12.77 | 13.15 | 13.37 | 10.54 | 12.63 |
| 12 March 19 | 13.02 | 13.17 | 12.65 | 14.12 | 12.74 | 14.55 | 12.24 | 13.76 | 11.56 | 15.62 | 13.83 | 13.94 |
| 03 May 19 | 19.21 | 19.57 | 16.45 | 18.97 | 16.68 | 19.29 | 14.60 | 20.28 | 16.75 | 20.18 | 21.69 | 18.73 |
| 22 June 19 | 24.46 | 18.37 | 21.97 | 31.91 | 23.72 | 21.32 | 25.93 | 24.66 | 28.58 | 22.16 | 24.62 | 25.15 |
| Overall mean | 16.38 | 17.53 | 16.61 | 17.53 | 16.73 | 18.73 | 17.14 | 18.90 | 18.42 | 19.33 | 18.70 | 18.64 |

**Principal component analyses (PCA) and redundancy analyses (RDA)**


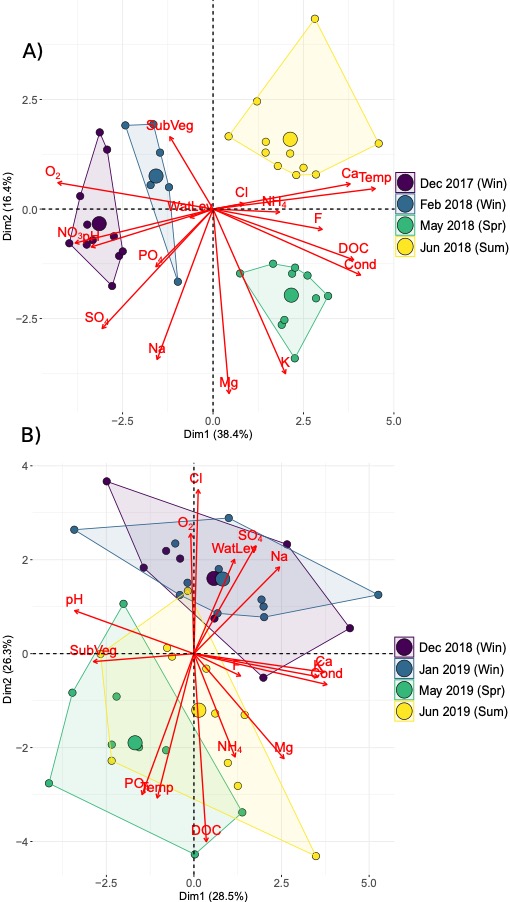


**Figure S9.** PCA biplots of ecosystem development and environmental conditions in the twelve FPMs for (A) winter 2017/2018 till summer 2018 and (B) winter 2018/2019 till summer 2019. Small dots represent FPMs and coloured polygons represent respective months and seasons (see legend for details), with larger dots representing the mean for a given month.


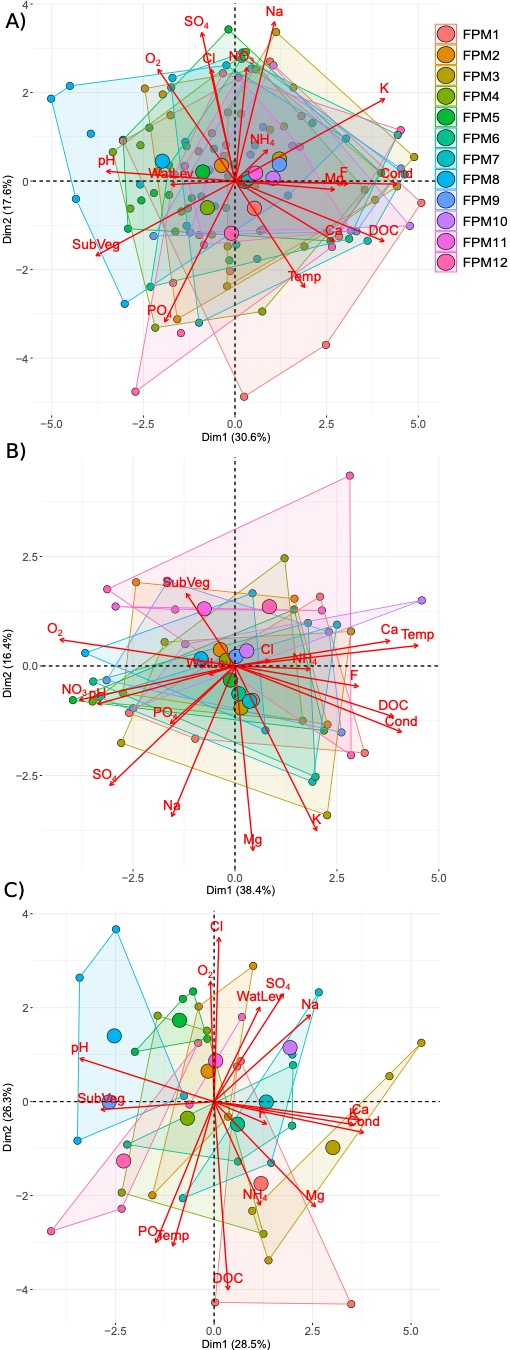


**Figure S10.** PCA biplots of environmental conditions during the individual seasons grouped by the twelve FPMs, with (A) showing available data for the entire study period (i.e., December 2018 – June 2019), (B) showing data for the first year (i.e., December 2018 – June 2019) and (C) showing data for the second year (i.e., December 2018 till June 2019) of ecosystem developments. Small dots represent individual months and coloured polygons represent respective FPMs (see legend for details), with larger dots representing the mean for a given FPM.

**Table S13.** Principal component (PC1, PC2) loadings for the principal component analysis.

| **PC1** | **Loading** | **PC2** | **Loading** |
| --- | --- | --- | --- |
| Specific conductivity | 0.91 | Sodium | 0.74 |
| Potassium | 0.84 | Sulfate | 0.69 |
| DOC | 0.84 | Nitrate | 0.53 |
| Fluoride | 0.64 | Chloride | 0.52 |
| Magnesium | 0.56 | Dissolved oxygen | 0.52 |
| Calcium | 0.56 | Potassium | 0.38 |
| Water temperature | 0.39 | DOC | - 0.28 |
| Sodium | 0.22 | Calcium | - 0.28 |
| Sulfate | - 0.19 | Submerged vegetation | - 0.35 |
| Water level | - 0.36 | Water temperature | - 0.50 |
| Phosphate | - 0.40 | Phosphate | - 0.66 |
| Dissolved oxygen | - 0.43 |  |  |
| pH | - 0.72 |  |  |
| Submerged vegetation | - 0.78 |  |  |

The following variables were excluded from principal component analyses (PCA) and redundancy analyses (RDA): Soil temperature, TOC, benthic macroinvertebrates, merolimnic insect emergence, zooplankton, tadpole coverage, crayfish and fish, terrestrial vegetation, ground beetles, leafhoppers, aquatic and terrestrial litter decomposition (see also methods section).

**Table S14.** Results of the redundancy analysis (RDA) for the entire study period (R^2^ adj. = 0.777).

|  | **Df** | **Variance** | **F** | **p adj. (Bonferroni)** |
| --- | --- | --- | --- | --- |
| Pond | 11 | 0.049 | 2.049 | 0.014 |
| Season | 2 | 0.013 | 3.103 | 0.017 |
| Time (month) from beginning | 1 | 0.02 | 9.198 | 0.003 |
| Residuals | 62 | 0.135 |  |  |

**Table S15.** Results of the redundancy analysis (RDA) for the first year of ecosystem development (R^2^ adj. = 0.556).

|  | **Df** | **Variance** | **F** | **p adj. (Bonferroni)** |
| --- | --- | --- | --- | --- |
| Season | 2 | 0.054 | 6.041 | 0.002 |
| Time (month) from beginning | 1 | 0.02 | 4.412 | 0.006 |
| Residuals | 36 | 0.162 |  |  |

**Table S16.** Results of the redundancy analysis (RDA) for the second year of ecosystem development (R^2^ adj. = 0.698).

|  | **Df** | **Variance** | **F** | **p adj. (Bonferroni)** |
| --- | --- | --- | --- | --- |
| Pond | 11 | 0.0869 | 3.639 | 0.003 |
| Season | 2 | 0.04345 | 10.003 | 0.003 |
| Time (month) from beginning | 1 | 0.00633 | 2.9153 | 0.023 |
| Residuals | 24 | 0.05212 |  |  |
